# A differentiation roadmap of murine placentation at single-cell resolution

**DOI:** 10.1038/s41421-022-00513-z

**Published:** 2023-03-17

**Authors:** Xiangxiang Jiang, Yue Wang, Zhenyu Xiao, Long Yan, Shanshan Guo, Yiming Wang, Hao Wu, Xuehan Zhao, Xiaoyin Lu, Hongmei Wang

**Affiliations:** 1 State Key Laboratory of Stem Cell and Reproductive Biology, Institute of Zoology, Chinese Academy of Sciences, Beijing, China; 2 NHC Key Laboratory of Study on Abnormal Gametes and Reproductive Tract, Anhui Medical University, Hefei, Anhui China; 3 Department of Obstetrics and Gynecology, the First Affiliated Hospital of Anhui Medical University, Hefei, Anhui China; 4 Institute for Stem Cell and Regeneration, Chinese Academy of Sciences, Beijing, China; 5 University of Chinese Academy of Sciences, Beijing, China; 6 Beijing Institute for Stem Cell and Regenerative Medicine, Beijing, China; 7 Department of Experimental Radiation Oncology, The University of Texas MD Anderson Cancer Center, Houston, TX USA

**Keywords:** Developmental biology, Bioinformatics

## Abstract

The placenta is one of the most important yet least understood organs. Due to the limitations of conventional research approaches, we are still far from a comprehensive understanding of mouse placentation, especially regarding the differentiation of trophoblast lineages at the early developmental stage. To decipher cell compositions and developmental processes, we systematically profile the single-cell transcriptomes of trophoblast cells from extraembryonic tissues (embryonic day 7.5 (E7.5) and E8.5) and placentae (E9.5–E14.5) at one-day intervals. We identify distinct trophoblast cell types during mouse placentation, including unreported progenitor cells and intermediate precursor cells. An updated differentiation roadmap of mouse trophoblast lineages is presented following systematic transcriptome analyses. Based on transcriptomic regulatory network inference, we specify transcription factors responsible for the regulation of dynamic developmental processes during lineage diversification. We map lineage differentiation trajectories and find that sinusoid trophoblast giant cells arise from the subpopulation of ectoplacental cone cells. We provide a comprehensive single-cell data resource to shed light on future mechanistic studies of the gene regulatory networks governing hemochorial placentation.

## Introduction

The placenta, a complex and organized supporting organ connecting the mother and fetus, guarantees the exchange of substances and hormone production for the successful maintenance of pregnancy^1^. It also serves as a formidable physical and immunological barrier, that protects the developing fetus from possible intrauterine infections^2^. Several landmark studies have highlighted the importance of the placenta for normal embryonic development, maternal health, and the long-term well-being of both individuals^3–5^. Malformation of the placenta may cause numerous placental deficiency-associated diseases, such as preeclampsia and intrauterine fetal growth restriction^1,6^. Both humans and mice display hemochorial placentation, which is characterized by the direct contact of the maternal vascular space with fetal trophoblast cells^7^. Accordingly, the mouse model has been a powerful tool for understanding human placental development and the etiology of pregnancy complications^8^. A recent study indicated that a considerable proportion of mutant mouse lines showing early embryonic lethality exhibit severe placental malformations, which emphasizes the vital role of the placenta during embryogenesis^9^.

The mouse cell lineage segregates into the inner cell mass (ICM) and the outer layer trophectoderm (TE) at the blastocyst stage (embryonic day 3.5, E3.5)^10–12^. Shortly after implantation (around E4.5), the mural TE differentiates into highly polyploid trophoblast cells, which are also known as primary parietal trophoblast giant cells (primary P-TGCs), through endoreplication^13,14^. In contrast, at the embryonic pole, the polar TE maintains its proliferative ability under the regulation of fibroblast growth factor 4 (*Fgf4*) secreted by the adjacent ICM. From E4.5 to E6.5, the undifferentiated polar TE forms the extraembryonic ectoderm (ExE)^15^. Both polar TE and ExE are considered to be sources of trophoblast stem cells (TSCs)^15–17^. The continuing expansion of the ExE gives rise to the ectoplacental cone (EPC). After gastrulation (E6.5–E8.5), the EPC cavity and the chorionic cavity (exocoelom) are formed^18,19^. The base of the ExE (EPC cavity) and the extraembryonic mesoderm together form the chorion plate, which makes attachment with the allantois at E8.5. The chorioallantoic fusion marks the beginning of hemochorial placentation^20,21^. Thereafter, chorionic trophoblast precursors differentiate into two layers of multinucleated syncytiotrophoblast cells (SynTI and SynTII) through cell-cell fusion, and a layer of mononucleated sinusoid TGCs (S-TGCs) forms the lining of the maternal blood space^22,23^. Together with endothelial cells lining the fetal blood vessels, these four layers of cells form the maternal-fetal interface of the labyrinth zone^24,25^. With the gradual establishment of the vascular network, EPC cells further differentiate into the spongiotrophoblast layer, which contains spongiotrophoblast cells (SpT), glycogen trophoblast (Gly-T) cells (which accumulate glycogen by E12.5), and several types of TGCs including spiral artery-associated TGCs (SpA-TGCs), canal TGCs (C-TGCs), and channel TGCs (Ch-TGCs)^26–29^. In the definitive placenta, a layer of P-TGCs (called secondary P-TGCs) lies between the spongiotrophoblast layer and the decidual tissue^3,30^. The spongiotrophoblast layer and secondary P-TGC layer are together referred to as the junctional zone. Trophoblast cells at the junctional zone are suggested to arise from the common EPC precursor^29,31^. However, the developmental histories of some trophoblast cell types remain unclear, including the origins of S-TGCs and secondary P-TGCs. Since the expression of *Ctsq* can only be detected after E12.5, the precursors of S-TGCs have not yet been identified^32^. Secondary P-TGCs are considered to arise from *Tpbpa* ^+∕−^ SpT precursor cells or EPC cells, but their exact developmental origin remains unknown^31,33^.

In recent decades, our knowledge about the developmental origins and physiological functions of mouse trophoblast lineages has significantly increased based on the use of mutant mouse lines and in situ labeling experiments^8^. In particular, a plethora of transcription factors (TFs) have been demonstrated to regulate trophoblast lineage differentiation^3^. For example, *Hand1* promotes the differentiation of TGCs^34^, *Ascl2* (*Mash2*) guarantees SpT maintenance^35^, and *Gcm1* drives chorioallantoic branching and labyrinth development^36^. However, the limited regulatory networks known to be involved in trophoblast lineage differentiation are insufficient to explain the dramatic developmental processes of the mouse placenta. In addition to this fragmented knowledge, we lack a systemic overview of the developmental regulation of mouse trophoblast cells from a single-cell transcriptomic perspective.

To address these questions, we exploited the single-cell RNA sequencing (scRNA-seq) technology to deconstruct the process of mouse placentation. We used several computational strategies combined with in situ hybridization to analyze and validate the gene expression patterns and developmental dynamics of mouse trophoblast cells. Collectively, our data provide a high-resolution single-cell transcriptome profile of the mouse placenta, and clarify important developmental trajectories of trophoblast cells, thus serving as a valuable resource for enriching our knowledge of the developmental process of hemochorial placentation.

### A single-cell resolution atlas of mouse trophoblast cells reveals developmental patterns of mouse placentation

**Fig. 1:**
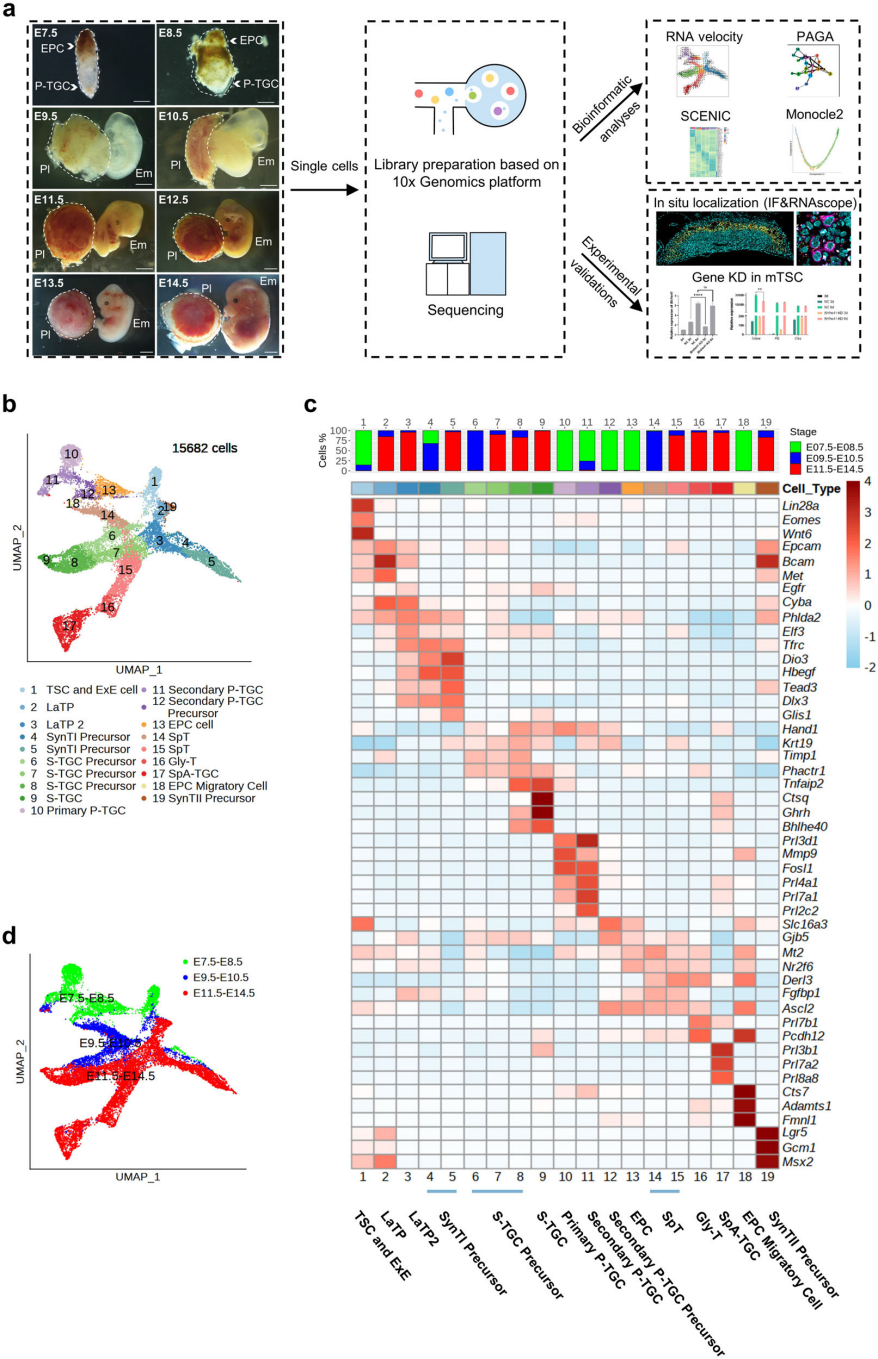
Single-cell resolution atlas of mouse trophoblast cells. **a** Schematic illustration of the strategies for the collection of single cells, transcriptome analyses and experimental validations used in this study. Only extraembryonic tissues and placentae within the area circled by dashed lines were digested to single cells. Left, representative images of sampled mouse EPC/placentae collected at the indicated time points. Scale bars: 500 μm (E7.5, E8.5); 1000 μm (E9.5, E10.5, E11.5); 2000 μm (E12.5, E13.5, E14.5). EPC ectoplacental cone, P-TGC parietal trophoblast giant cell, Pl placenta, Em embryo. **b** Expression matrix-based uniform manifold approximation and projection (UMAP) plot showing all the mouse trophoblast cells (15,682 cells). Cells are colored by cell clusters after the unsupervised clustering step. **c** Heatmap for the main cell types depicting the expression of differentially expressed genes. Representative highly expressed genes of each cell type are displayed in the right panel. The bar plots in the top panel show the stage distribution for each cell type. The color key from light blue to red indicates low to high gene expression level. **d** UMAP plot as shown in (**b**), with cells colored by merged time points of sample collection.

To decipher the temporal transcriptional landscape of hemochorial placentation, we collected mouse extraembryonic tissues (E7.5 and E8.5) and placentae (E9.5, E10.5, E11.5, E12.5, E13.5, and E14.5) representing diverse developmental time points associated with dramatic morphological changes. The collected samples were pooled together at each time point for single cell suspenstion preparation. We then performed scRNA-seq by using the 10× Genomics Chromium system (Fig. 1a)^37^. After excluding the cells with fewer than 500 genes, 39,603 single cells were filtered (a median of 3430 genes were detected in each cell; Supplementary Fig. S1a). Unsupervised clustering using the Seurat package identified 14 broad cell clusters annotated by transcriptional signatures (Supplementary Fig. S1b, c; Table S1).

Trophoblast cells are building blocks of the functional placenta and can be easily distinguished from other cell types at the maternal‒fetal interface based on the expression of cytokeratins (Supplementary Fig. S1c, d). After further excluding multiple and low-quality cells from clusters A to C (*Krt8*^+^), a total of 15,682 mouse trophoblast cells passed strict filtering for further analyses (Supplementary Fig. S 1e, f, methods). All of these cells were identified and annotated in 19 clusters based on previously defined marker genes (Fig. 1b, c and Supplementary Fig. S2a–d, Table S2), including TSCs and ExE cells (cluster 1, *Lin28a*^+^ and *Eomes*^+^ ^38,39^), labyrinth trophoblast progenitor cells (LaTP, cluster 2, *Epcam*^+^ and *Met*^+^ ^40,41^, LaTP2, cluster 3, *Epcam*^+^ and *Egfr*^+^ ^41^), SynTI precursor cells (clusters 4 and 5, *Tfrc*^+^ ^42^), S-TGCs (cluster 9, *Ctsq*^+^ ^32^), primary P-TGCs (cluster 10, *Prl3d1*^+^ ^28^, and *Ugcg*^+^ ), secondary P-TGCs (cluster 11, *Prl3d1*^+^ , and *Prl2c2*^+^ ^33,43,44^), SpT cells (clusters 14 and 15, *Ascl2*^+^ ^35^), glycogen trophoblast cells (cluster 16, *Prl7b1*^+^ , and *Pcdh12*^+^ ^26,27^), and SynTII precursor cells (cluster 19, *Gcm1*^+^ ^45,46^). Cluster 17 cells were suggested to be a mixed population of SpA-TGCs, channel TGCs, and canal TGCs based on the expression of *Prl3b1* and *Prl8a8*, since no exclusive markers have been reported to differentiate these three types of TGCs until now^28,33^. Additionally, cell clusters 6–8 were suggested to be S-TGC precursor cells which were *Timp1*^+^, *Hand1*^+^, *Prl3d1*^–^, *Prl3b1*^–^, and *Ctsq*^–^. Furthermore, at the early developmental stages (E7.5–E8.5), we identified EPC cells (cluster 13), EPC migratory cells (cluster 18), and secondary P-TGC precursor cells (cluster 12) which were characterized in subsequent analyses. Overall, we captured most of the reported trophoblast cell types during mouse placentation except for syncytiotrophoblast cells, which may not have been identified because of technical difficulties in handling fragile multinucleated cells.

According to the uniform manifold approximation and projection (UMAP) plot, we noticed that trophoblast cells fell into three communities, E7.5–E8.5, E9.5–E10.5, and E11.5–E14.5 (Fig. 1d), which indicated that mouse placentation might go through 3 distinct developmental stages over time. The demarcations between these three stages were chorioallantoic fusion (E8.5–E9.5) and the formation of a mature placental structure (E10.5–E11.5).

### The development of mouse trophoblast cells at E7.5 and E8.5

**Fig. 2:**
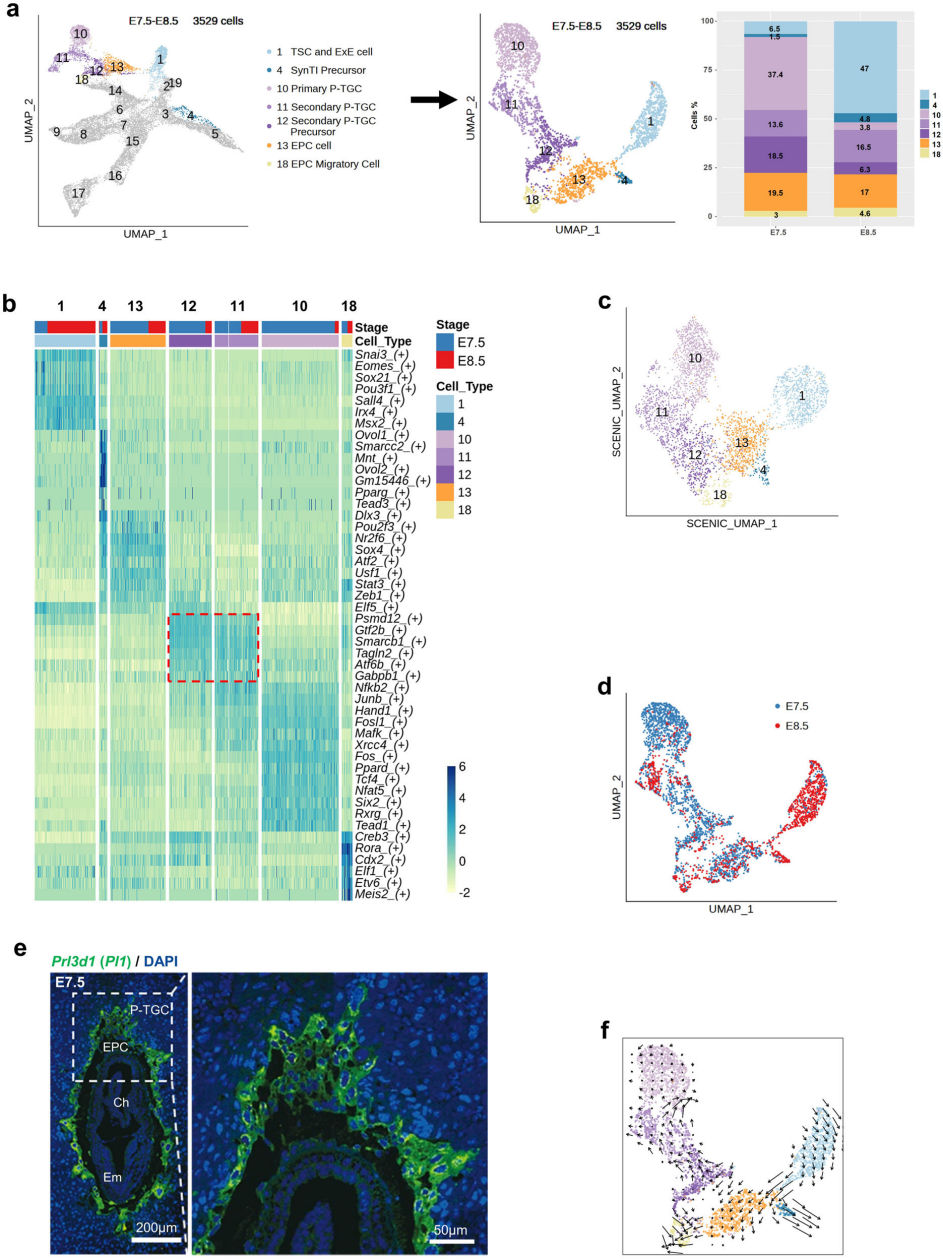
Analysis of E7.5 and E8.5 mouse trophoblast cells. **a** UMAP plot showing trophoblast cells of E7.5 and E8.5, with cells colored by cell clusters from Fig. 1b (left panel); UMAP plot reproduced with E7.5–E8.5 trophoblast cells by the Seurat flow (middle panel); bar plots showing the cell percentages of trophoblast cell clusters in E7.5 and E8.5 (right panel). **b** Heatmap showing the activities of representative TFs in E7.5 and E8.5 mouse trophoblast cells. The color key from yellow to blue indicates low to high TF activity. Cells are colored by cell clusters and sampling time points at the top of the heatmap. **c** Regulon matrix-based UMAP plot showing the cells and cell clusters in the right panel of (**a**). **d** UMAP plot as shown in the middle panel of (**a**), with cells colored by sampling time points. **e** A representative section of an E7.5 mouse embryo immunostained with *Prl3d1* (green). Nuclei were stained with DAPI (blue). Scale bar is shown as indicated. P-TGC parietal trophoblast giant cell, EPC ectoplacental cone, Ch chorion, Em embryo. **f** The RNA velocity field is projected onto the UMAP plot shown in the right panel of (**a**), and the outlier cells between cluster 12 and cluster 13 were excluded. Arrows show the local average velocity evaluated on a regular grid.

To gain a deep understanding of lineage differentiation at the early developmental stage, we separately analyzed trophoblast cells from E7.5 and E8.5 (Fig. 2a), which mainly contained cell clusters 1, 4, 10, 11, 12, 13, and 18.

As interpreted above, we determined the cell identities of TSC and ExE cell (cluster 1), SynTI precursor (cluster 4), primary P-TGC (cluster 10), and secondary P-TGC (cluster 11). However, the cell types of clusters 12, 13, and 18 needed to be further annotated. Although the gene expression patterns between cluster 12 and cluster 11 (secondary P-TGCs) were quite different (Supplementary Fig. S3a), they shared many exclusively expressed TFs (Fig. 2b, indicated by the red frame), including the chromatin remodeling gene *Smarcb1* and the epithelial cell differentiation-associated gene *Tagln2*, according to the SCENIC (single-cell regulatory network inference and clustering) analysis (Fig. 2b, c). This indicated that cluster 12 cells presented a closer relationship with secondary P-TGCs. In addition, secondary P-TGCs were more abundant at E8.5 than at E7.5 (Fig. 2a (right), d), indicating that the population of secondary P-TGCs enlarged upon chorioallantoic fusion, whereas primary P-TGCs were more abundant at E7.5 than at E8.5 (Fig. 2a (right), d, and e). Moreover, RNA velocity analysis showed that cluster 12 cells could differentiate into secondary P-TGCs (Fig. 2f). Therefore, cluster 12 cells were annotated as secondary P-TGC precursor cells. EPC cells were known to be derived from TSCs and ExE cells^31^, and cluster 13 cells and cluster 4 SynTI precursor cells were indicated to be differentiated from TSCs and ExE cells according to the RNA velocity results (Fig. 2f), thus cluster 13 cells were suggested to be EPC cells. In addition, we found a new migratory trophoblast cell cluster (cluster 18) with high expression of *Inhbb*, *Fmnl1*, *Pcdh12*, and *Tpbpa* (Supplementary Fig. S3b). These cells might be the reported *Pcdh12* ^+^ cells located outside of the embryo, along the uterine crypt^26^. The RNA velocity results suggested that these cells were differentiated from EPC cells (cluster 13) and secondary P-TGC precursor (cluster 12) cells (Fig. 2f). Gene Ontology (GO) analysis showed that highly expressed genes in cluster 18 cells were mainly enriched in cell migration and vasculature development (Supplementary Fig. S3c, d; Table S3), which illustrated that cluster 18 may consist of a group of unknown EPC migratory cells promoting angiogenesis.

Additionally, to further understand critical TFs that played roles during trophoblast differentiation at E7.5 and E8.5, the SCENIC analysis was performed (Fig. 2b, c). The observed TF activities and gene expression patterns showed that during the differentiation of primary and secondary P-TGCs, TFs such as, *Hand1*^34,47^*, Junb*^48^, and *Xrcc4* were specifically active (Fig. 2b and Supplementary Fig. S3e). *Psmd12* and *Gtf2b* might be involved in the differentiation of secondary P-TGCs (Fig. 2b). *Eomes*^39^, *Pou3f1*, and *Irx4* might contribute to the maintenance of TSCs and ExE cells (Fig. 2b and Supplementary Fig. S3e). In addition, SynTI precursor cells and EPC cells shared some TFs such as *Dlx3*^49^, *Pou2f3*, and *Sox4*^50^ (Fig. 2b and Supplementary Fig. S3e). However, other TFs such as *Ovol2*, *Tead3*^51^, and *Pparg* were specifically activated in SynTI precursor cells (Fig. 2b and Supplementary Fig. S3e). The emergence of EPC migratory trophoblast cells might be driven by *Cdx2*^52^ and *Rora* (Fig. 2b and Supplementary Fig. S3e).

To have a perception about critical signaling pathways for TSC and ExE differentiation, the cell-cell communication analysis was performed with all cell types at E7.5 and E8.5 (Supplementary Fig. S4a). The results indicated that BMP (BMP2, BMP4, and BMP7), FGF (FGF3, FGF5, and FGF10), and integrin (a1b1, a3b1, and a6b1, etc) signaling pathways might play important roles during TSC and ExE cell differentiation (Supplementary Fig. S4b).

### The cell fates of S-TGC and SpT are determined before chorioallantoic fusion (E8.5)

**Fig. 3:**
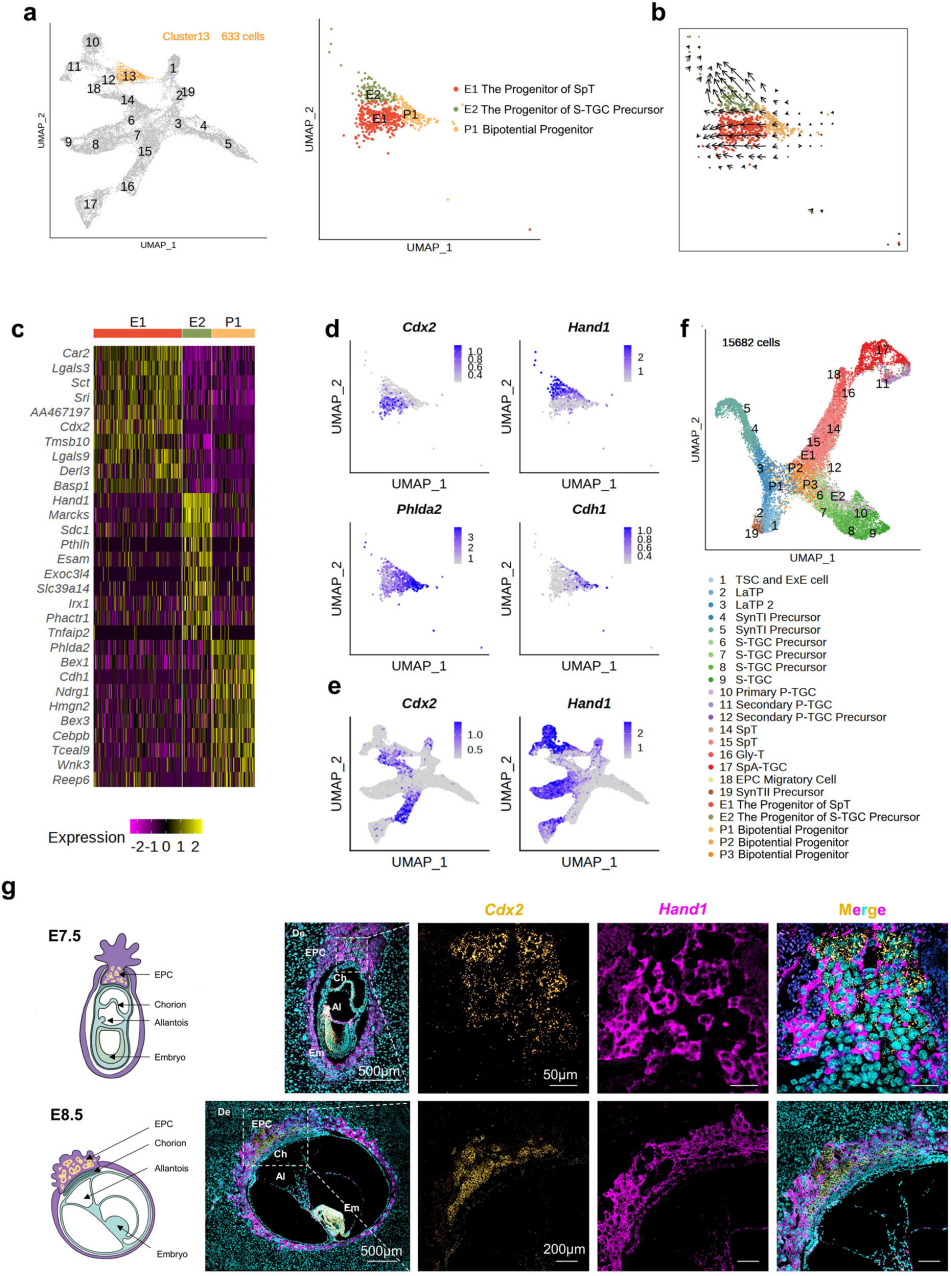
Analysis of mouse EPC cells (cluster 13). **a** UMAP plot showing EPC cells, colored by cell clusters from Fig. 1b (left panel). UMAP plot separated from the left panel, with cells colored by sub-clusters (right panel). **b** RNA velocity field projected onto the right panel of (**a**). **c** Heatmap showing top 10 highly expressed genes for each cell cluster shown in the right panel of (**a**). Yellow corresponds to a high expression level; purple and black correspond to low expression levels. **d** UMAP plots as shown in the right panel of (**a**), showing the expression of *Cdx2*, *Hand1*, *Phlda2*, and *Cdh1*. The color key from grey to blue indicates low to high gene expression level, here and after. **e** UMAP plots as shown in Fig. 1b, showing the expression of *Cdx2* and *Hand1*. **f** UMAP plot showing 15,682 mouse placental trophoblast cells after the integration analysis of single-cell data from different sampling time points, with cells colored by cell clusters. **g** Representative images of E7.5 and E8.5 mouse placenta sections probed for *Cdx2* (yellow) and *Hand1* (magenta) transcripts. Nuclei were stained with DAPI (cyan), here and after. Scale bars are shown as indicated. De decidua, EPC ectoplacental cone, Ch chorion, Al allantois, Em embryo.

EPC cells can further differentiate into SpT cells and various trophoblast giant cells^33^, indicating that progenitor cells with different potentials reside in the EPC region. To identify progenitor cells within EPC, we performed a separate analysis for cluster 13 cells (Fig. 3a). Interestingly, unsupervised clustering and RNA velocity analyses (Fig. 3a, b) showed that EPC cells could be divided into clusters P1, E1 and E2 cells, and showed two differentiation potentials. E1 cells expressed *Cdx2* (Fig. 3c, d and Supplementary Table S4), which was also expressed by SpT cells (Fig. 3e), and SpT cells were differentiated from EPC cells^28,53^. Thus, we suggested that E1 cells were the progenitors of SpT cells. E2 cells expressed *Hand1* (Fig. 3c, d), which was highly expressed by S-TGC precursor cells (Fig. 3e). Therefore, E2 cells might be the progenitors of S-TGC precursor cells, then cluster E2 was annotated as the progenitor of S-TGC precursor. The integration analysis results indicated that cluster E1 cells were integrated with SpT cells, and that cluster E2 cells were integrated with S-TGC precursor cells (Fig. 3f), further confirming our E1 and E2 annotations. The RNA velocity results also indicated that P1 cells could differentiate into E1 and E2 cells (Fig. 3b), thus *Phlda2*^high^, *Cdh1*^+^, *Cdx2*^–^, and *Hand1*^–^ P1 cells were annotated as EPC bipotential progenitor cells (Fig. 3d). To further confirm whether progenitors of SpT (cluster E1) and progenitors of S-TGC precursor (cluster E2) were derived from EPC, we performed the RNA in situ hybridization assay in EPC tissues at E7.5 and E8.5 (Fig. 3g), and the results indicated that cells of both clusters E1 (*Cdx2*^+^) and E2 (*Hand1*^+^) were found in the EPC region. Since the progenitor cells of SpT cells and S-TGC precursors have emerged before E8.5, the cell fates of SpT and S-TGC could be determined before chorioallantoic fusion.

After the identification of P1 bipotential progenitor cells at the root of cluster E1 (the progenitor of SpT) and E2 (the progenitor of S-TGC precursor), we then examined whether bipotential progenitor cells existed at the root of SpT cells (clusters 14 and 15) and S-TGC precursor cells (clusters 6 and 7) after chorioallantoic fusion (E8.5). After further unsupervised clustering of clusters 6, 7, 14, and 15 cells (Supplementary Fig. S5a), additional cell clusters (P2 and P3) were found, and these cells were also *Phlda2*^high^*, Cdh1*^+^, *Cdx2*^–^, and *Hand1*^–^ (Supplementary Fig. S5b, c), similar to the characteristics of P1 cells. And clusters P1, P2, and P3 cells were distributed together during the integration analysis (Fig. 3f), further reflecting their homologies. Then, clusters P2 and P3 cells, which were also located at the root of SpT cells and S-TGC precursor cells in E9.5–E14.5 placentae (Fig. 1d and Supplementary Fig. S5b, c), were annotated as bipotential progenitor cells that could differentiate into S-TGC precursor cells and SpT cells after E8.5.

### Differentiation trajectories of mouse trophoblast cells at the branch level

**Fig. 4:**
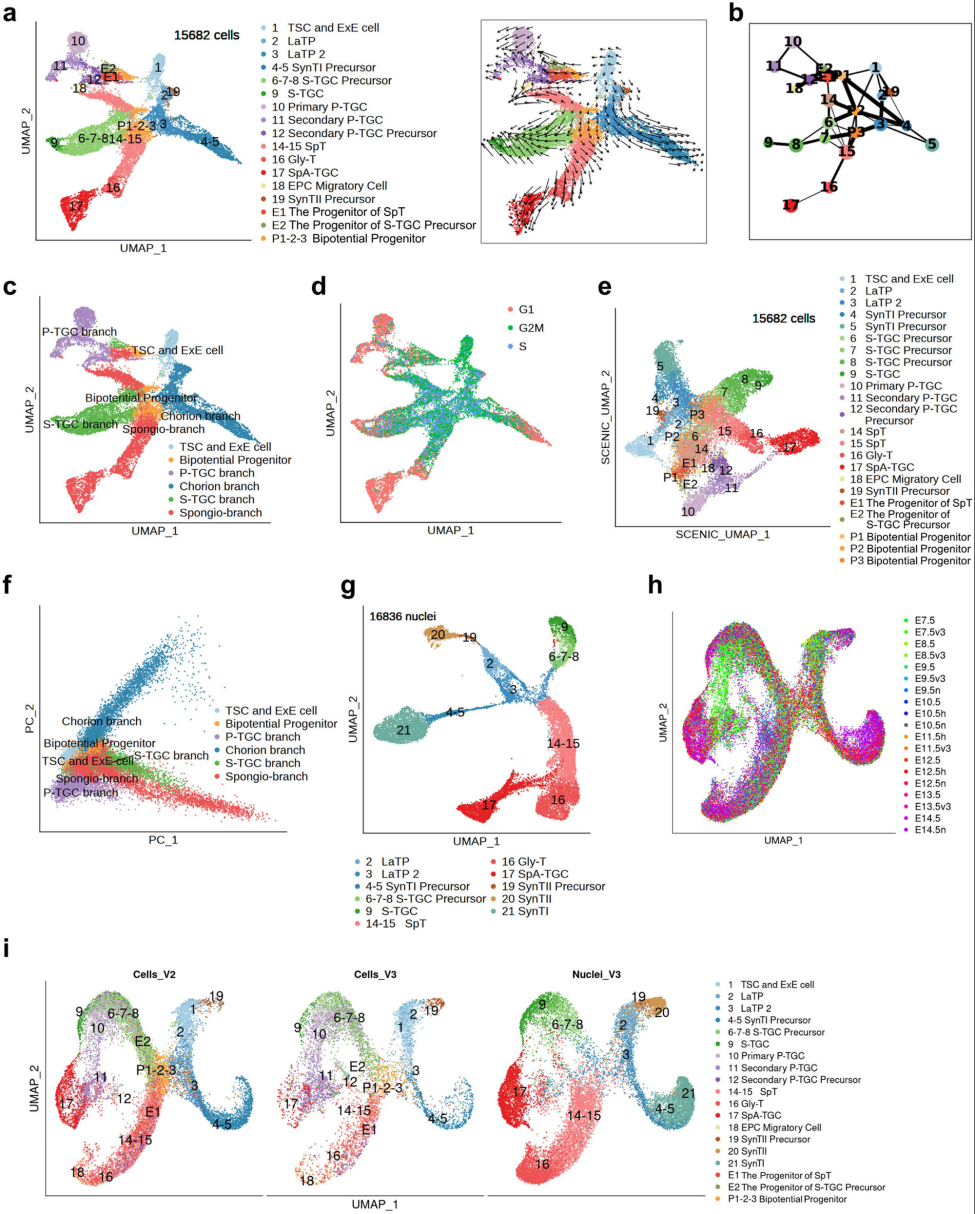
Differentiation trajectories of mouse trophoblast cells at the branch level. **a** UMAP plot as shown in Fig. 1b, with cells colored by cell clusters as indicated (left panel). The RNA velocity field is projected onto the left UMAP plot after excluding cells with the percent.mt value below 1%, and excluding genes with multiple rate kinetics. Arrows show the local average velocity evaluated on a regular grid (right panel). **b** Partition-based graph abstraction (PAGA) summarizing the relationships between the cell clusters as shown in (**a**). Nodes correspond to the cell clusters (larger nodes indicate more cells), and edges reflect the confidence of adjacency between clusters (thicker edges indicate higher confidence). **c** UMAP plot as shown in (**a**), with cells colored by the cell type at the branch level. **d** UMAP plot as shown in (**a**), with cells colored by cell cycle phases. **e** Regulon matrix-based UMAP plot showing the cells and cell clusters in (**a**). **f** The principal component analysis (PCA) plot showing all the mouse trophoblast cells, with cells colored by cell branches shown in (**c**). **g** UMAP plot showing nuclei of mouse trophoblast cells that have been reported^41^. **h** UMAP plot showing 10× v2 15,682 mouse placental trophoblast cells, 10× v3 8067 mouse placental trophoblast cells, and 16836 mouse placental trophoblast nuclei illustrated by the integration function of Seurat, with cells and nuclei colored by the sample name. Sample names without suffix letters indicate 10× v2 single-cell samples; Sample names with letter h indicate the 10× v2 single-cell samples from another study of us (GSE152903) associated with placental hematopoiesis; Sample names with v3 indicate 10× v3 single-cell samples; Sample names with letter n indicate the 10× v3 single nuclei samples. **i** UMAP plot as shown in (**h**) split by data origin, with cells (left and middle) and nuclei (right) colored by cluster identities.

To reconstruct the developmental histories of mouse trophoblast lineages from a holistic perspective, we mapped the differentiation trajectories for the 15,682 mouse trophoblast cells by using RNA velocity (Fig. 4a) and Partition-based Graph Abstraction (PAGA) (Fig. 4b) analyses. From TSCs to terminally differentiated cells, the roadmap of trophoblast lineage diversification had multiple branching points. We combined previous annotations to define four developmental branches: P-TGC branch, chorion branch, S-TGC branch, and spongio-branch (Fig. 4c). The P-TGC branch included primary P-TGCs, secondary P-TGC precursor cells, and secondary P-TGCs. The chorion branch was initiated from LaTP cells and terminally differentiated into syncytiotrophoblast cells. The S-TGC branch contained S-TGCs and their precursor cells. The spongio-branch mainly consisted of SpT, Gly-T, and maternal blood vessel-associated TGCs (including SpA-TGC, C-TGC, and Ch-TGC). In addition, according to cell cycle analysis (Fig. 4d), stem cells and precursor cells were mainly in G2M or S phase, and terminally differentiated cells were mainly in G1 phase, corresponding well with the differentiation trajectories of mouse trophoblast cells. Interestingly, the S-TGC branch and spongio-branch were both derived from bipotential progenitor cells (Fig. 4a-c).

The PAGA (Fig. 4b) and SCENIC (Fig. 4e) results indicated that the S-TGC branch and spongio-branch shared the same initiation point (clusters P2 and P3), although they were on two distinct differentiation paths, further indicating their same developmental origin. It was previously thought that S-TGCs arose from *Tpbpa*^*−*^ precursor cells, which could originate from either the EPC, chorion, or both^29,30,33,54^. Our results indicated that S-TGCs were differentiated from the subpopulation of EPC cells (progenitors of S-TGC precursor, cluster E2, *Hand1*^+^) before E8.5 (Fig. 3), and continued to be differentiated from bipotential progenitor cells (clusters P2 and P3) after E8.5 (Supplementary Fig. S5), but were not derived from the chorion cells. Thus, S-TGCs and chorion-derived syncytiotrophoblast cells originated from completely different progenitor cells, which was further supported by the principal component analysis (PCA) results (Fig. 4f), though S-TGCs and chorion-derived syncytiotrophoblast cells both resided in the placental labyrinth.

To further confirm the main findings about mouse trophoblast development, additional biological replicates of mouse extraembryonic tissues and placentae (E7.5, E8.5, E9.5, E11.5, and E13.5) were subjected to scRNA-seq experiments with 10× v3 kits (Supplementary Fig. S6a–e). After selecting 8067 10× v3 mouse trophoblast cells with high quality, the main structure of dimensionality reduction and trophoblast cell types were recapitulated through further mapping and annotating of these single-cell data with 15,682 10× v2 mouse trophoblast cells (Supplementary Fig. S6d), reflecting the reliability of previous results.

To further investigate the developmental trajectories of these four developmental branches, we integrated our single-cell data (10× v2 and v3) with the recently reported single nuclei RNA sequencing (snRNA-seq) data of mouse placental labyrinth, which significantly supplemented the information of syncytiotrophoblast cells (Fig. 4g-i)^41^. Meanwhile, progenitors of SpT (cluster E1), progenitors of S-TGC precursor (cluster E2), and bipotential progenitor cells (clusters P1, P2, and P3) were also distributed as expected according to the integration analysis (Figs. 3f, 4i). Overall, the distributions of singlecells and singlenuclei were as expected, except for P-TGCs, which were integrated with either S-TGCs or SpA-TGCs, possibly because of the similarities of TGCs themselves. There were almost no P-TGCs in the snRNA-seq dataset, which could have been due to the sampling time, and P-TGCs were mainly sequenced in E7.5 and E8.5 in our single-cell dataset (Fig. 1d). The structure of the trophoblast branches was optimized and enriched with the help of snRNA-seq data of SynTI and SynTII, and our single-cell data were indicated to supply more cells at the root of the S-TGC branch and the spongio-branch (Fig. 4i).

### Parallel differentiation of SynTI and SynTII from labyrinth trophoblast progenitor cells

**Fig. 5:**
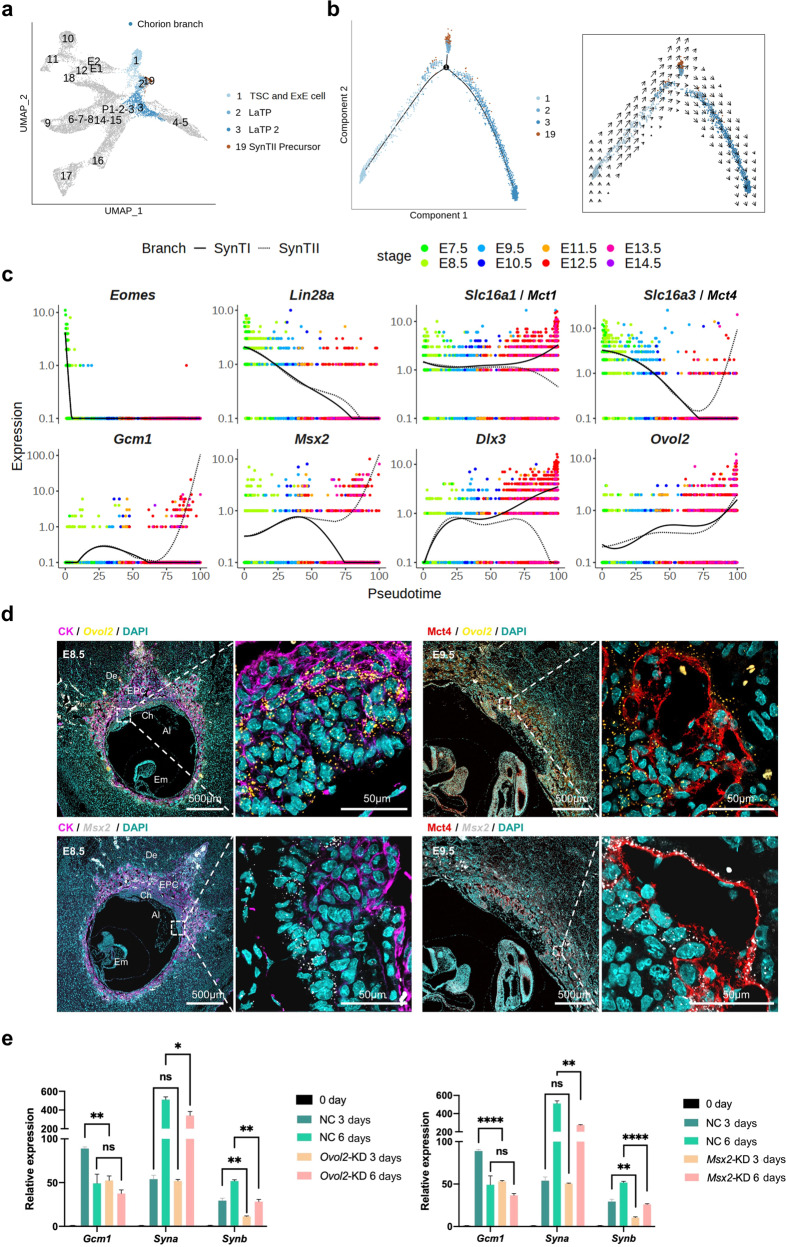
Establishment of the chorion branch. **a** UMAP plot showing chorion branch, with cells colored by cell clusters as indicated. **b** Pseudotime ordering of cells as shown in (**a**), with cells colored by cell clusters (left). The RNA velocity field is projected onto the pseudotime trajectory plot shown in the left panel (right). **c** Pseudotemporal kinetics plots showing the expression of TFs involved in the development of chorion branch cells. As shown in the top-left corner of the image, the solid line indicates the dynamic expression of TFs in SynTI branch cells across developmental pseudotime; and the dashed line indicates the dynamic expression of TFs in SynTII branch cells across developmental pseudotime. Points are colored by the sampling time as indicated on the top-right corner of the image. **d** Representative images of E8.5 and E9.5 mouse placenta sections probed for *Ovol2* (yellow) and *Msx2* (gray) transcripts, and co-stained with cytokeratin (CK, magenta). Scale bars are shown as indicated. De decidua, EPC ectoplacental cone, Ch chorion, Al allantois, Pl placenta, Em embryo. **e** Statistical RT-qPCR results for the expression of *Gcm1*, *Syna*, and *Synb* in *Ovol2*–KD and *Msx2*–KD mTSCs compared to the negative controls (NC, here and after) during mouse trophoblast cell differentiation. Data are displayed as the mean ± SEM of three independent replicates (*P < 0.05, **P < 0.01, ****P < 0.0001, ns not significant, Student’s *t* test) in mTSCs during mouse trophoblast cell differentiation.

To understand the establishment of the maternal-fetal interface in the labyrinth, we analyzed the developmental process of the chorion branch in detail (Fig. 5a). Pseudotime and RNA velocity analyses of our chorion branch single-cell data (clusters 1 to 3, and 19) showed that both LaTP2 cells (which contributed to SynTI) and SynTII precursor cells differentiated from LaTP cells, which were derived from TSCs and ExE cells (Fig. 5b). To supplement the information available for terminally differentiated syncytiotrophoblast cells, we also checked recently reported snRNA-seq data (Supplementary Fig. S7a)^41^. The pseudotemporal ordering of the snRNA-seq data reconfirmed that SynTII arose from LaTP via SynTII precursor cells. While, LaTP2 cells were derived from LaTP cells and further contributed to SynTI via SynTI precursor cells (Supplementary Fig. S7b, c).

To avoid interfering with cells on other branches, we reproduced the UMAP plot by using only the chorion branch cells (Supplementary Fig. S8a). The RNA velocity analysis and PCA reconfirmed the developmental trajectories that we defined (Supplementary Fig. S8b, c). To pinpoint critical TFs that regulate the development of the functional exchange interface, we performed the SCENIC analysis for the chorion branch cells and corresponding nuclei (Supplementary Fig. S8d, e). The expression of stemness maintenance-associated factors, such as *Eomes* and *Lin28a*, gradually decreased during the developmental process (Fig. 5c). *Gcm1*, *Tbx3*, and *Cebpa* were involved in the formation of SynTII as expected^36,55^, and *Msx2*, *Hes7*, and *Hoxc13* were also suggested to play roles during SynTII differentiation (Fig. 5c and Supplementary Figs. S7d, S8e). *MSX2* was reported to be involved in the invasion of trophoblast cells and served as a repressor of the human syncytiotrophoblast lineage^56^. *Hes7* could be induced by Notch pathway activation^57^. *Hoxc13* mutant mice and *HOXC13* mutant patients showed hair defects^58^. *Dlx3*^49,59^, *Rb1*^60^, *Glis1*^41^*, Pax2*^41^, and *Tead3*^51^ were reported to drive the differentiation of SynTI, and our data suggested that *Ovol2* played similar roles (Fig. 5c and Supplementary Figs. S7d, S8e–g). Additionally, *Ovol2* has been reported to play roles in vascular angiogenesis during early embryogenesis^61^.

RNA in situ hybridization illustrated that *Ovol2* and *Msx2* were expressed as early as E8.5, and they were distributed in the chorion region (Fig. 5d). Considering their continuous expression during placentation (Supplementary Fig. S7e), *Ovol2* and *Msx2* might play indispensable roles during the differentiation of mouse syncytiotrophoblast. To further verify their regulatory function during the syncytialization process, we performed gene knockdown (KD) of *Ovol2* and *Msx2* in mouse TSCs (mTSCs) when the differentiation process started after the withdrawal of recombinant human FGF4 and heparin (Supplementary Fig. S8h), and the expressions of downstream lineage marker genes were quantified by RT-qPCR (Fig. 5e). During the differentiation process, mTSCs could differentiate to SynT I/II, SpA-TGCs, S-TGCs, Gly-T cells, etc^16^. *Ovol2*-KD and *Msx2*-KD mTSCs exhibited a significant decrease in syncytialization-related marker genes, such as *Gcm1*, *Syna*, and *Synb*, during in vitro differentiation compared to negative control (NC) cells (Fig. 5e), which further reflected the regulatory functions of *Ovol2* and *Msx2* during mouse trophoblast syncytialization.

### Differentiation of S-TGCs from the subpopulation of EPC cells

**Fig. 6:**
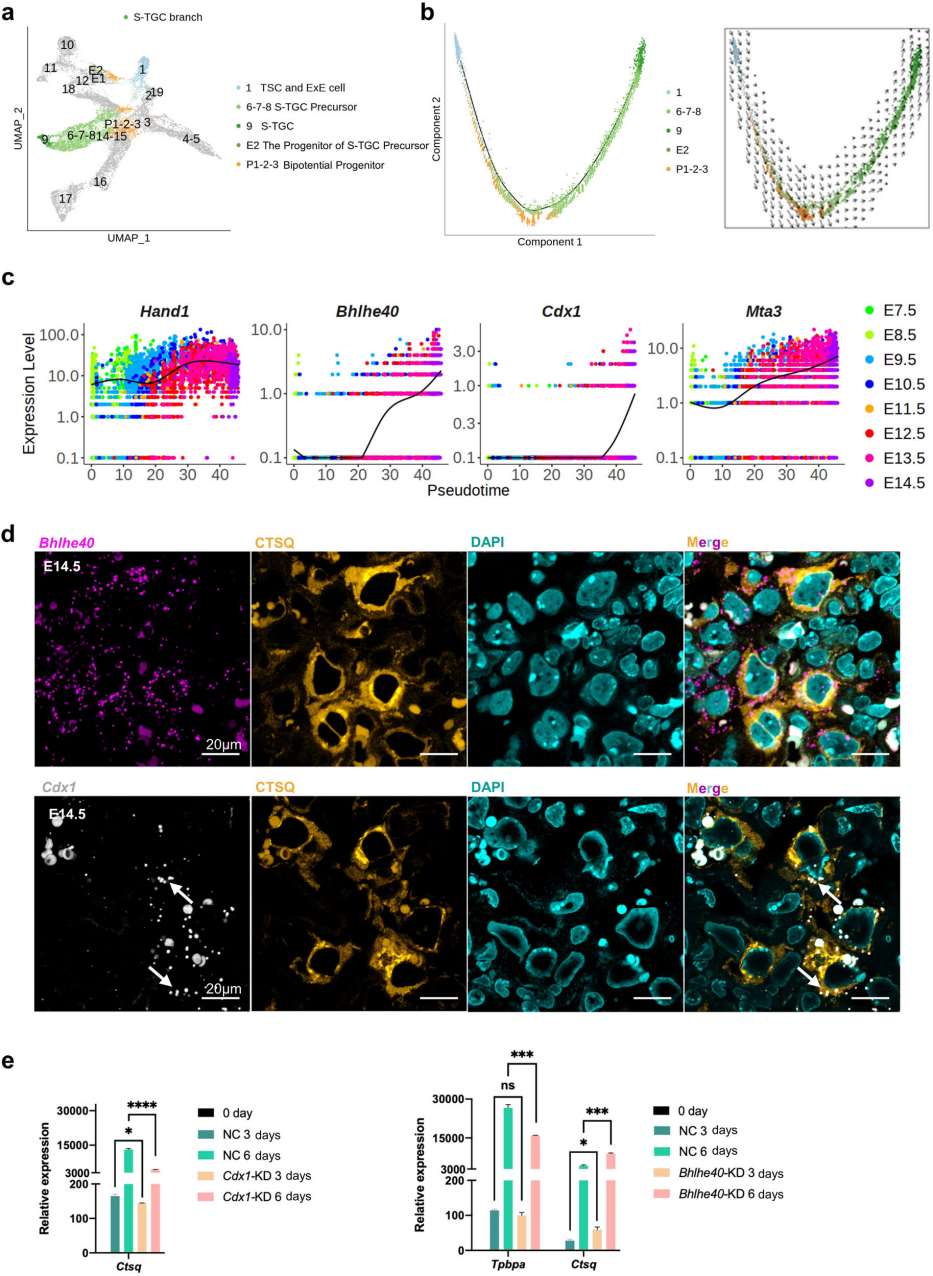
Differentiation of S-TGCs from the subpopulation of EPC cells. **a** UMAP plot showing the S-TGC branch, with cells colored by cell clusters as indicated. **b** Pseudotime ordering of cells as shown in (**a**), with cells colored by cell clusters (left). The RNA velocity field is projected onto the pseudotime trajectory plot shown in the left panel (right). **c** Pseudotemporal kinetics plots showing the expression of TFs involved in the development of S-TGC branch cells. The solid line indicates the dynamic expression of TFs in S-TGC branch cells across developmental pseudotime. Points are colored by sampling time as indicated on the right of the image. **d** Representative images of E14.5 mouse placenta sections probed for *Bhlhe40* (magenta) and *Cdx1* (gray) transcripts, and co-stained for cathepsin Q (CTSQ, yellow). White arrows point to merged signals of *Cdx1* and CTSQ. Scale bars are shown as indicated. **e** Statistical RT-qPCR results for the expression of *Ctsq* in *Cdx1*-KD mTSCs and statistical RT-qPCR results for the expression of *Tpbpa* and *Ctsq* in *Bhlhe40*-KD mTSCs, compared to the negative controls during mouse trophoblast cell differentiation. Data are displayed as the mean ± SEM of three independent replicates (*P < 0.05, ***P < 0.001, ****P < 0.0001, ns not significant, Student’s *t* test).

The data obtained from our molecular trajectory analysis suggested that S-TGCs were originated from the EPC subpopulation (cluster E2, the progenitor of S-TGC precursor) rather than the chorion lineage (Fig. 4a, b). To further decipher the developmental process of S-TGCs and verify our previous interpretations, TSCs and ExE cells (cluster 1), bipotential progenitor cells (clusters P1, P2, and P3), the progenitor of S-TGC precursor (cluster E2), and other S-TGC branch trophoblast cells (clusters 6 to 9) were subjected to further analyses (Fig. 6a and Supplementary Fig. S9a). The RNA velocity and PCA results (Supplementary Fig. S8b, c) indicated that TSCs and ExE cells could differentiate into EPC bipotential progenitors (cluster P1), which would further contribute to S-TGC branch cells via the subpopulation of EPC cells (cluster E2). In addition, S-TGC branch cells were suggested to arise from both early (cluster P1) and late (clusters P2 and P3) stage bipotential progenitors (Supplementary Fig. S9b, c). In line with the RNA velocity results, pseudotime analysis indicated the differentiation of S-TGC branch cells from bipotential progenitor cells (clusters P1, P2, and P3) (Fig. 6b and Supplementary Fig. S9d). We then performed SCENIC analysis to infer the activity of TFs that may regulate the development of S-TGC branch trophoblast cells (Supplementary Fig. S9e). *Hand1* was reported to be closely related to the differentiation of trophoblast giant cells^47,62^, and *Hand1* continued to be active along the differentiation trajectory of S-TGC branch cells as expected (Fig. 6c). In addition to *Hand1*, the TFs *Bhlhe40*/*Stra13*, *Mta3*, and *Cdx1* were also suggested to be involved in the differentiation of S-TGCs (Fig. 6c and Supplementary Fig. S9e, f).

In support of these findings, RNA in situ hybridization showed that *Bhlhe40* and *Cdx1* were expressed in CTSQ^+^ S-TGCs (Fig. 6d). The cytosolic CTSQ signals marked the distribution of S-TGCs, and the transcripts of *Bhlhe40* and *Cdx1* were mostly distributed in the cytoplasm of S-TGCs. Cytosolic *Cdx1* signals were indicated by the overlapping signals of CTSQ and *Cdx1* (indicated by white arrows in Fig. 6d). To further verify their regulatory function during the differentiation of S-TGCs, we performed gene knockdown (KD) of *Cdx1* and *Bhlhe40* (Supplementary Fig. S9g) and observed the dynamic changes of TGC-related marker genes in mTSC in vitro differentiation system (Fig. 6e). *Cdx1*-KD mTSCs showed relative downregulation of *Ctsq* expression during S-TGC differentiation (Fig. 6e), which provided additional evidence for the involvement of *Cdx1* in the specialization of S-TGCs. However, *Bhlhe40*-KD only reduced the expression of *Tpbpa*, which was a differentiation marker of SpA-TGCs, and the expression of *Ctsq* increased (Fig. 6e). This indicated that *Bhlhe40* might play an inhibitory role during the differentiation of S-TGCs.

### Parallel differentiation of Gly-T cells and SpA-TGCs from SpT cells

**Fig. 7:**
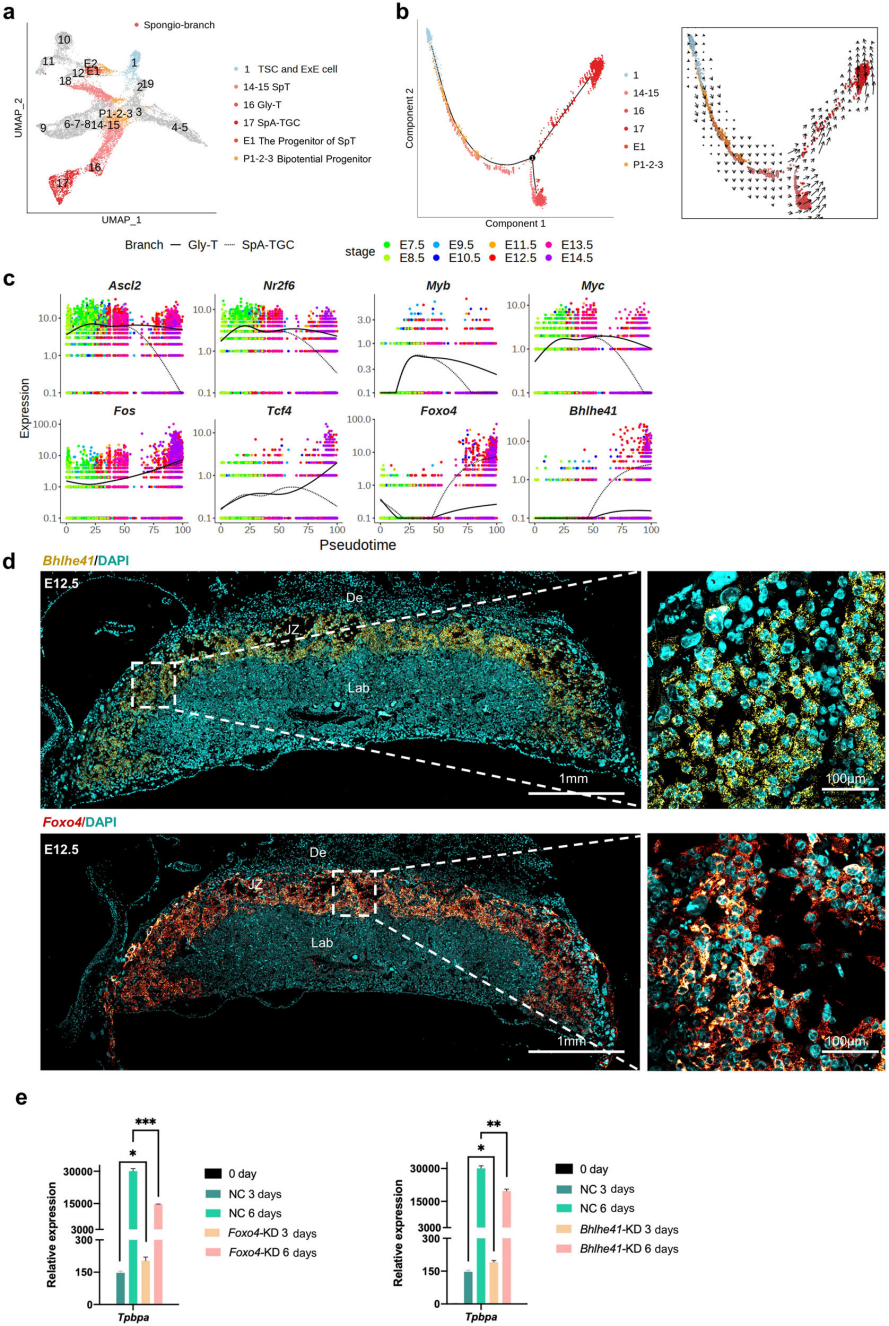
Parallel differentiation of Gly-T cells and SpA-TGCs from SpT cells. **a** UMAP plot showing spongio-branch cells, with cells colored by cell clusters as indicated. **b** Pseudotime ordering of cells as shown in (**a**), with cells colored by cell clusters (left). The RNA velocity field is projected onto the pseudotime trajectory plot shown in the left panel, after excluding cells with the percent.mt value below 1%, and excluding genes with multiple rate kinetics (right). **c** Pseudotemporal kinetics plots showing the expression of TFs involved in the development of spongio-branch cells. As shown in the top-left corner of the image, the solid line indicates the dynamic expression of TFs in Gly-T branch cells across developmental pseudotime, and the dashed line indicates the dynamic expression of TFs in SpA-TGC branch cells across developmental pseudotime. Points are colored by the sampling time, as indicated in the top-right corner of the image. **d** Representative images of E12.5 mouse placenta sections probed for *Bhlhe41* (yellow) and *Foxo4* (red) transcripts. Scale bars are shown as indicated. De decidua, JZ junctional zone, Lab labyrinth. **e** Statistical RT-qPCR analysis of *Tpbpa* expression in *Foxo4*-KD and *Bhlhe41*-KD mTSCs compared to the negative controls during mouse trophoblast cell differentiation. Data are displayed as the mean ± SEM of three independent replicates (*P < 0.05, **P < 0.01, ***P < 0.001, Student’s *t* test).

To verify the differentiation trajectory of spongio-branch trophoblast cells, TSCs and ExE cells (cluster 1), EPC bipotential progenitor cells (cluster P1), the progenitor cells of SpT (cluster E1), late-stage bipotential progenitor cells (clusters P2 and P3), SpT cells (clusters 14 and 15), Gly-T cells (cluster 16), and SpA-TGCs (cluster 17) were selected for further analyses (Fig. 7a and Supplementary Fig. S10a). The differentiation trajectories indicated by the RNA velocity results suggested that the progenitor cells of SpT (cluster E1) that arose from EPC bipotential progenitors (cluster P1) could differentiate into SpT cells (Supplementary Fig. S10b). Corresponding to the PCA results (Supplementary Fig. S10c), pseudotime trajectory results further indicated that SpA-TGCs and Gly-T cells were in two parallel differentiation directions of SpT cells (Fig. 7b and Supplementary Fig. S10d). SCENIC analysis (Supplementary Fig. S10e) showed that *Ascl2* (*Mash2*) and *Cdx2* were involved in the maintenance of the spongio-branch trophoblast cells as previously reported^53,63^. In addition, we identified some other TFs that played similar roles to *Ascl2*, such as the nuclear receptor *Nr2f6* and the proto-oncogene *Myc* (Supplementary Fig. S10e). Meanwhile, TFs *Fos* and *Tcf4* might boost the specification of glycogen trophoblast cells. After E12.5, *Foxo4* and *Bhlhe41* might be activated for the differentiation of SpA-TGCs (Fig. 7c and Supplementary Fig. S10e-g).

Moreover, in situ hybridization showed that transcripts of *Foxo4* and *Bhlhe41* were highly enriched in the junctional zone where SpA-TGCs reside (Fig. 7d). To further verify their regulatory function during the differentiation of SpA-TGCs, we performed gene knockdown (KD) of *Foxo4* and *Bhlhe41* in mTSCs (Supplementary Fig. S10h). The expression level of *Tpbpa* was decreased after 6 days of mTSC in vitro differentiation (Fig. 7e), which indicated that *Foxo4* and *Bhlhe41* were likely to be involved in the formation of the spongiotrophoblast layer and differentiation of SpA-TGCs.

## Discussion

**Fig. 8:**
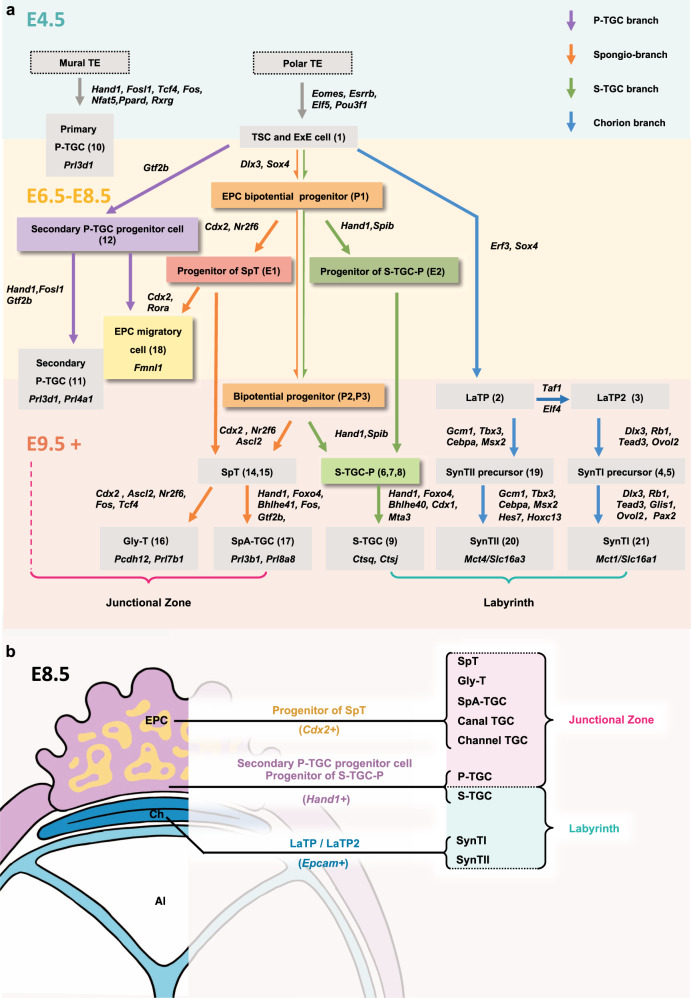
Differentiation roadmap of mouse trophoblast cell lineage illustrated with bioinformatic evidence. **a** The mural TE differentiates into primary P-TGCs as previously reported^28^. The polar TE can give rise to TSCs which are common progenitors of LaTP cells, EPC cells, and secondary P-TGC cells. Secondary P-TGCs are differentiated from TSCs and ExE cells via secondary P-TGC progenitor cells. The S-TGC branch and spongio-branch have the same progenitor cells, and their fates are determined when the EPC structure forms (before chorioallantoic fusion). SpA-TGCs and Gly-T cells are both differentiated from SpT. TFs associated with each developmental process are indicated beside the corresponding arrow. The cell clusters with colored backgrounds (except for the gray background) were newly identified. The number in parentheses next to the cell type represents the cell cluster. The markers of terminally differentiated cell types are listed below the corresponding cell types. TE trophectoderm, TSC trophoblast stem cell, ExE extraembryonic ectoderm, EPC ectoplacental cone, SpT spongiotrophoblast, LaTP labyrinth trophoblast progenitor, P-TGC parietal trophoblast giant cells, Gly-T glycogen trophoblast, SpA-TGC spiral artery-associated trophoblast giant cells, S-TGC sinusoid trophoblast giant cells, S-TGC-P sinusoid trophoblast giant cell precursor, SynT I/II syncytiotrophoblast layer I/syncytiotrophoblast layer II. **b** The EPC contains heterogenous trophoblast progenitor cells. *Cdx2*^+^ EPC cells are progenitors of spongiotrophoblast cells, glycogen trophoblast cells, and SpA-TGC, canal TGC, and channel TGC within the junctional zone. *Hand1*^+^ EPC cells and secondary P-TGC progenitor cells give rise to S-TGC and secondary P-TGC, respectively. LaTPs (*Epcam*^+^) from the chorion are the developmental origin of two layers of syncytiotrophoblast cells. Ch chorion, Al allantois.

Due to the lack of documentation of the entire dynamic placentation, the mechanisms underlying mouse placental development remain unclear. Previous studies of mouse placenta using scRNA-seq only sequenced placental cells at a single developmental time point^64–66^. Although a recent study of snRNA-seq covered multiple developmental time points, it mainly focused on the specialization of the syncytiotrophoblast in the labyrinth^41^. In this study, we provided the first profile of the main processes of placental trophoblast differentiation at single-cell resolution covering the most vital stages of mouse placentation. Notably, we identified lineage progenitor cells and intermediate precursor cells on different developmental branches, especially before chorioallantoic fusion. We obtained a high-resolution lineage tree (Fig. 8a) of mouse trophoblast cells, which offered a global overview of trophoblast lineage differentiation histories. TFs highly relevant to differentiation trajectories were comprehensively annotated, enriching the differentiation roadmap as a valuable resource for studying the regulation of the hemochorial placentation.

P-TGCs are considered as a vital cell type during trophoblast lineage differentiation, contributing to successful embryonic implantation and pregnancy maintenance^31,33^. Secondary P-TGCs are located between the maternal decidual and junctional zone. The origin of secondary P-TGCs has long been a mystery, and previous studies have reported that secondary P-TGCs arose from *Tpbpa* ^+∕−^ SpT precursor cells or EPC cells^33^. Here, we have identified the progenitor cells (cluster 12) of secondary P-TGCs, demonstrating that secondary P-TGCs were not derived from SpT precursor cells (Fig. 8a, b). In the future, the developmental trajectory of secondary P-TGC could be better confirmed by the precise localization of secondary P-TGC precursor cells and lineage tracing experiments.

The densely organized labyrinth structure gradually forms shortly after the chorioallantoic fusion. S-TGCs in the labyrinth zone directly connect with maternal blood, playing an essential role in substance exchange and hormone regulation^28^. Thus, decoding the developmental origin of S-TGCs is particularly important. Previous studies indicated that *Tpbpa*^–^ precursor cells, which may reside in either the EPC, chorion, or both, could give rise to S-TGCs^29,30,33,54^. We showed that S-TGC branch trophoblast cells were only derived from a subpopulation of EPC cells, which excluded the possibility that S-TGC originated from the chorion cells. Furthermore, we revealed that *Hand1* and *Cdx1* were involved in the differentiation of S-TGC branch cells (Fig. 8a). Although S-TGCs bathe in the labyrinth zone, and was originally thought to be from LaTP cells, LaTP cells could not develop into all labyrinth trophoblast lineages, and only contribute to syncytiotrophoblast bilayers. The selected TFs responsible for the differentiation of each type of TGC could serve as good candidates for decoding their cell fate decisions and physiological functions (Fig. 8a).

A systematic understanding of the differentiation process of mouse trophoblast cells has long been lacking. Most terminally differentiated mouse trophoblast cells have been studied for gene expression patterns and physiological functions. However, there is still a lack of understanding of the progenitor and precursor cells between stem cells and terminally differentiated cells. Our systematic analysis identified the bipotential progenitor cells and progenitors of S-TGC and SpT in the EPC (Fig. 8b). The mechanisms that determine the cell fates of these progenitor cells need further investigation.

Overall, we presented a high-resolution roadmap of mouse trophoblast lineage differentiation and identified regulatory molecules of the dynamic placentation, which could be a valuable resource for future studies of hemochorial placentation and pregnancy maintenance.

## Materials and methods

### Animals and collection of placentae

All animal experiments were approved by the Animal Ethics Committee and the Institutional Animal Care and Use Committee, Institute of Zoology, Chinese Academy of Sciences. Wild-type C57BL/6 J mice (6–8 weeks old) were purchased from SPF Biotechnology (Beijing). The mice were all housed at the experimental animal center of the Institute of Zoology, Chinese Academy of Sciences, under controlled 12-h light/dark cycles (temperature 20–22 °C; humidity 30%–70%). All animals were provided ad libitum access to a regular rodent chow diet. Females were paired with fertile males at 6 P.M. When a copulation plug was present on the following morning (before 10 A.M.), that time point was recorded as embryonic day 0.5 (E0.5). We collected extraembryonic tissues at E7.5 and E8.5 and placentae at E9.5, E10.5, E11.5, E12.5, E13.5, and E14.5, respectively. Placentae were also collected at E10.5, E11.5, and E12.5 in another study (GSE152903) of us^67^. Extraembryonic tissues/placentae between E8.5 and 14.5 were obtained from a single pregnancy, and extraembryonic tissues were obtained at E7.5 from two pregnancies when the cells from one pregnancy did meet the minimum sample size. All pregnant mice passed a strict selection process based on physiological conditions. Samples from a single pregnancy were used to avoid subtle variations in developmental phases between different mice. Detailed sample information is provided in Supplementary Fig. S2c.

### Isolation of mouse placental singlecells

The extraembryonic tissues (E7.5 and E8.5) or placentae (E9.5–E14.5) collected from one pregnant mouse were pooled together, completely cut into tiny pieces and rinsed 3 times with DMEM/F-12 1:1 cell culture medium (11320082, Gibco). The extraembryonic tissue (E7.5 and E8.5) pieces were digested with TrypLE™ Express Enzyme (1×, 12605010, Gibco) for 20 min at 37 °C. The placental tissue pieces were digested with collagenase Type IV (0.5 mg/mL, C5138, Sigma‒Aldrich) and DNase I (0.2 mg/mL, DN25, Sigma‒Aldrich) for 20 min at 37 °C. The released extraembryonic cells (E7.5 and E8.5) and placental cells were filtered through 40 µm cell strainers (352340, Falcon). To exclude the remaining red blood cells, the filtered cells were lysed with red blood cell lysis buffer [155 mM NH_4_Cl (A9434, Sigma‒Aldrich), 10 mM KHCO_3_ (237205, Sigma‒Aldrich), 0.1 mM EDTA (E6758, Sigma‒Aldrich)] for 3 min. To exclude cell debris and adherent cells, the released placental cells were centrifuged at 1200× *g* for 15 min after loading these cells onto a Percoll gradient consisting of 28% and 60% Percoll (17-0891-01, GE Healthcare), and only cells that were sedimented between 28% and 60% Percoll were collected. All purified single cells were suspended in DMEM/F12 cell culture medium for the following procedures.

### Preparation of the scRNA-seq library and sequencing

Single-cell libraries were constructed using the 10× Single-cell 3ʹ Library & Gel Bead Kit v2 and v3 according to the manufacturer’s protocol^37^. Briefly, cell counts were assessed with a hemocytometer (Luna-fl, Logos Biosystems), and the cell concentration was adjusted to 1000 cells/µL. A total of 10,000 cells were added to each channel, and approximately 5000 cells were then captured. Each single-cell library was constructed independently. Captured cells were lysed, and the released RNA was barcoded through reverse transcription from individual gel beads in emulsion. The cDNA was then amplified for the library construction, and the qualities of cDNA and cDNA libraries were assessed with an Agilent 2100 system. Finally, the libraries were sequenced on an Illumina HiSeq X Ten platform (Novogene, Beijing). Every single-cell library was sequenced individually in a single lane, generating approximately 120 GB of raw data. The sampling of extraembryonic tissues and placentae was performed on 8 different days, and the single-cell libraries were prepared and sequenced in 11 batches (Supplementary Fig. S2c).

### 10× genomics data pre-processing

The samples labeled E10.5 h, E11.5 h, and E12.5 h were from our previous study (Supplementary Fig. S1a, GSE152903)^67^. The mouse strain (C57BL/6 J) and the single-cell method (10× Single-cell 3ʹ Library & Gel Bead Kit v2) were the same as in this study. The raw fastq files of 10× v2 in this study and the other study (GSE152903) were processed with Cell Ranger 2.1.1, and the raw fastq files of 10× v3 in this study were processed with Cell Ranger 7.0.0, with the default mapping arguments^37^. Reads were mapped to the mouse mm10 genome and counted with GRCm38.89 annotation. Next, the cellranger aggr command was executed to adjust the sequencing depth of different 10× v2 samples (including the samples of GSE152903) to the same level, and the mean number of reads per cell was greater than 25,000 post normalization. The sequencing depth of 10× v3 samples was adjusted to 32,000 reads per cell post normalization.

### Dimension reduction and clustering

The dimension reduction and clustering procedures were performed with the Seurat v3.1.5 R package^68^. Briefly, after excluding doublets and low-quality cells, as stated in the “Selection of mouse trophoblast cells with high quality” section below, when creating Seurat objects with count matrixes, only those genes that were expressed in more than three cells were retained. Then, the count matrix was normalized by library-size correction using the default scale factor of 10,000. Highly variable genes were calculated by the FindVariableFeatures function with the ‘mean.var.plot’ method. After scaling the normalized data with highly variable genes, the top 50 principal components (PCs) were computed with the RunPCA function. Uniform Manifold Approximation and Projection (UMAP) was performed to project cells to 2 dimensions using the RunUMAP function. During this process, dimensions were selected according to the obvious inflection point in the elbow plot, and the “correlation” metric and the “umap-learn” method were used. The shared nearest neighbor was found by using the FindNeighbors function with dimensions used to construct the UMAP plot, and the unsupervised clustering based on the Louvain algorithm, as implemented in the FindClusters function, was performed to identify cell clusters. The resolution parameter for identifying clusters was tuned so that the number of clusters produced was large enough to capture most of the biological variability. After each round of clustering, we plotted a heatmap for highly expressed genes of all cell clusters. If cell clusters were intermingled or there were almost no differences between any two cell clusters in the heatmap, the clustering resolution needed to be downregulated. If the differences between any two cell clusters in the heatmap were very significant, we tried a higher resolution parameter until no difference between any two cell clusters emerged. The resolution for the clustering of all 39,603 10× v2 cells was 0.05 (Supplementary Fig. S1b); the resolution for the clustering of 15,682 trophoblast cells was 0.6 (Fig. 1b); the resolution for the further clustering of cluster 13 EPC cells was 0.2 (Fig. 3a, right); and the resolution for the further clustering of clusters 6, 7, 14, and 15 cells was 0.4 (Supplementary Fig. S4a).

### Selection of mouse trophoblast cells with high quality

The filtered expression matrix with cell barcodes and gene names of 10× v2 single-cell data was loaded by the Read10× function of the Seurat v3.1.5 R package^68^. Firstly, the cells with the percentage of mitochondrial genes (percent.mt) below 10% and the number of detected genes (nFeature_RNA) above 500 were retained to exclude apoptotic or dead cells (Supplementary Fig. S1a). The samples labeled E10.5 h, E11.5 h, and E12.5 h were from another study (GSE152903) of us^67^. The expression matrix and metadata of each sample were exported and then loaded to the Python environment, and Scrublet package was used to determine doublet or multiplet cells with customized parameters according to the recommended multiplet rate reference table from 10× Genomics^37,69^. Next, the Seurat objects of different samples were created independently with an expression matrix and metadata containing cell barcodes and multiple cell information, and these Seurat objects were merged to generate the UMAP plot (Supplementary Fig. S1b). An unsupervised clustering method was used to divide different cell types (Supplementary Fig. S1c). These cell types were mainly annotated according to their highly expressed genes and the published annotations of cells at the mouse maternal-fetal interface^64,66,67^. Firstly, *Krt8*^+^ cells (clusters A to C) were placental trophoblast cells (Supplementary Fig. S1d, e). Among these cells, the *Prl3b1*^+^ cluster A cells were SpA-TGCs; *Phlda2*^+^ *Prl3d1*^–^ cluster B cells were TSC and ExE derived trophoblast cells. *Prl3d1*^+^ cluster C cells were P-TGCs. *S100g*^+^ *Gkn2*^+^ cluster D cells were primitive endoderm cells. *Afp*^+^ *Apoa1*^+^ *S100g*^+^ cluster E cells were yolk sac epithelial cells. *Sox11*^+^ *Igfbp2*^+^ cluster F and J cells were suggested to be cells contaminated from mouse embryos during the primary cell isolation, and the *Pou5f1*^+^ cluster F cells were the embryo stem cells. The *Mdk*^+^ cells were embryo stromal cells. *Prl8a2*^+^ *Dcn*^+^ cluster G cells were decidual stromal cells. *Acta2*^+^ *Dcn*^+^ cluster H cells were decidual pericytes. *Kdr*^+^ *Pecam1*^+^ cluster I cells were endothelial cells. *Ptprc*^+^ *Cd74*^+^ cluster K cells were decidual immune cells. *Ppbp*^+^ *Pf4*^+^ cluster L cells were megakaryocytes. *Alas2*^+^ *Hba-x*^+^ cluster M cells were erythrocytes. *Myb*^+^ *Mpo*^+^ cluster N cells were suggested to be hematopoietic cells. Considering the larger size of trophoblast cells compared to immune cells and endothelial cells, we further labeled the trophoblast cells with the nFeature_RNA value below 2000 and the percent.mt value below 1% as low-quality trophoblast cells to further exclude apoptotic trophoblast cells and trophoblast nuclei, except for SpA-TGCs and P-TGCs (clusters 1 and 3 in Supplementary Fig. S1e). We expected that many TGCs would be captured as nuclei, with fewer detected genes and low levels of mitochondrial genes for their giant nuclei. Therefore, multiplet cells and low-quality trophoblast cells were excluded from the following analyses, and only those genes that were expressed in more than three cells were retained. After stringent filtering, 15,682 mouse trophoblast cells were retained (Supplementary Fig. S2a, b, detailed information is provided in Supplementary Fig. S2c). The procedure of selecting mouse trophoblast cells with high quality for 10× v3 single-cell data was similar to that applied for the 10× v2 single-cell data, and finally 8067 mouse trophoblast cells were retained (Supplementary Fig. S5).

### The computing of highly expressed genes and GO analysis

We computed the highly expressed genes of each cell cluster by using the FindAllMarkers function of Seurat v3.1.5 R package^68^. The base of natural logarithm and the Wilcoxon Rank Sum test were used as defaults. The genes that were detected in a minimum fraction were 0.25, and the log fold change threshold was 0.25 by default. The heatmap was plotted based on the top 10 highly expressed genes (according to p.val. adjust and fold change values) of each cell cluster by using the DoHeatmap function of Seurat (Fig. 3c and Supplementary Fig. S3a) or based on representative highly expressed genes of each cell cluster by using the pheatmap v1.0.12 R package after computing the mean expression levels (scale data) of each cell cluster (Fig. 1c and Supplementary Fig. S1c). To do the Gene Ontology (GO) enrichment for cell clusters of E7.5 and E8.5, the highly expressed genes of cell clusters from E7.5 and E8.5 were used as the input for the compareCluster function of the clusterProfiler v3.18.1 R package^70^; ontology was set as “BP”; pAdjustMethod was set as “BH”; pvalueCutoff was set as 0.0001. Then, the comparing image was plotted with the dotplot function. To show the GO terms for EPC migratory cells (cluster 18) with related genes, the highly expressed genes of cluster 18 were used as the input for the enrichGO function of clusterProfiler with the same settings as the compareCluster function, and the GO network image was plotted with the cnetplot function.

### PAGA analysis

The Seurat object was converted to a loom file by the as.loom function of the loomR v0.2.1.9000 R package^68^, and the loom file was then loaded to the Python environment by using the sc.read_loom function of the Scanpy v1.6.1 Python package^71^. After setting the neighbor argument, the relationship between different cell clusters was evaluated by the partition-based graph abstraction (PAGA) analysis with the Scanpy Python package^71^.

### Pseudotime analysis

The Monocle2 v2.14.0 R package^72^ was used to compute the developmental pseudotimes of mouse trophoblast cells. After selecting the trophoblast cells of individual branches, the UMI count data and metadata were exported from the Seurat object. A new Seurat object was built based on the UMI count data and metadata, and only those genes that were expressed in more than three cells were retained. The Seurat object was then converted to the Monocle2 object by the importCDS function of Monocle2^72^. The genes selected for the unsupervised ordering of the cells were based on the adjustment of the value of dispersion and mean expression. We chose the dispersion value of 1 and the mean expression value of 0.01 to perform the following pseudotime analysis with default settings of the Monocle2 R package^72^.

### RNA velocity analysis

Read annotations for mouse placenta samples were obtained using the velocyto.py (v0.17.17) command-line tool (velocyto run10×) with BAM, genome annotation, and repeat annotation files^73^. The BAM file was produced by using the default parameters of the Cell Ranger software (10× Genomics)^37^. The GRCm38.89 genome annotations from the Cell Ranger pre-built references were used to count molecules while separating them into three categories: ‘spliced’, ‘unspliced’, and ‘ambiguous’. Repeat annotation files were downloaded from the UCSC genome browser. The velocyto.R package v0.6 was used to calculate RNA velocity values for selected genes from each cell^73^, and highly variably expressed genes, as computed by the FindVariableFeatures function of Seurat, were further filtered based on cluster-wise expression (with the thresholds of unspliced = 0.1 and spliced = 0.5). The remaining highly variably expressed the genes selected were as the input for velocyto.R. Finally, RNA velocity vectors were embedded to the UMAP plot produced by the Seurat R package^68^ and the pseudotime plot that was generated by the Monocle2 R package^72^. When computing the velocity vectors for datasets containing SpA-TGCs (Fig. 4a (right), 7b (right) and Supplementary Fig. S10b), the cells with the percent.mt value below 1% were first excluded, and the genes with multiple rate kinetics^74^ were also computed and excluded.

### The mapping and annotating of 10× v3 single-cell data

Firstly, the “RunUMAP” procedure with “return.model = TRUE” was applied to the 10× v2 single-cell data with the Seurat 4.0.0 R package^75^. Then, the anchors between the 10× v2 and 10× v3 single-cell data were identified with the “FindTransferAnchors” function (reference.reduction = “pca”, dims = 1:30), and the “MapQuery” function (reference.reduction = “pca”, reduction.model = “umap”) was used to project the 10× v3 single-cell data onto the 10× v2 single-cell data based UMAP structure. Meanwhile, the cell types of 10× v3 single-cell data were annotated by the cell types of 10× v2 single-cell data appropriately (Supplementary Fig. S5c, d).

### Integrated analysis of scRNA-seq and single nuclei RNA sequencing data

The integration analysis was performed with either 10× v2 15,682 trophoblast cells alone (Fig. 3f), or 10× v2 15,682 trophoblast cells, 10× v3 8067 trophoblast cells, and 16,836 trophoblast nuclei^41^ by using the Seurat v3.1.5 R package (Fig. 4h, i)^68^. Firstly, the single cells of different batches were merged. After renormalizing the Seurat object, we selected highly variably expressed genes based on the mean.var.plot method at the FindVariableFeatures step. Then, these feature genes and default 30 dimensions of canonical correlation analysis (CCA) were analyzed with FindIntegrationAnchors, IntegrateData and RunPCA, etc. Before the FindIntegrationAnchors step, the Seurat object was split by sampling time points of different origins to exclude the differences between different samples. The tree number was 50 as default when finding integration anchors. Subsequently, the Seurat pipeline was executed for the dimensionality reduction. Then, UMAP plots were split by datasets and presented with the corresponding original cell labels (Fig. 4i).

### SCENIC analysis

SCENIC analysis was carried out following the SCENIC (v0.11.0) command line protocol^76^. The SCENIC command line version was used to perform gene regulatory network inference, regulon prediction, and cellular enrichment (Area Under the Curve, AUC) processes with trophoblast cells from E7.5–E14.5, E7.5–E8.5, the sinusoid branch, and the spongio-branch, respectively. After the integration analysis of our 10× v2 single-cell data with the reported single nuclei data^41^, the scale data of chorion branch cells and nuclei with all filtered genes were used to perform the SCENIC analysis. The SCENIC UMAP was computed based on the AUC matrix. Regulon specificity scores (RSS) were computed based on the cell clusters identified by Seurat. Finally, the SCENIC AUC heatmap was plotted with the reported regulons and representative top regulons of each cell cluster using the heatmap R package. To construct the transcriptional regulatory network between transcriptional regulators and targets for the development of specific trophoblast types, the representative TFs and corresponding targets with high weights (importance > 10) were exported from the list of adjacencies computed by the “arboreto_with_multiprocessing.py” command with the corresponding trophoblast cells. Then the filtered TFs and targets were imported to the Cytoscape (v3.6.0) software to construct a regulatory network. In the network, the red nodes indicated the TFs, and the light green nodes indicated the targets. Node size indicated the number of connections, and the line width indicated the weight of a connection.

### The cell-cell communication analysis for TSCs and ExE cells

The cell-cell communication analysis for TSCs and ExE cells was performed with all kinds of cell types at E7.5 and E8.5 by CellPhoneDB v2.1.7^77^, as TSCs and ExE cells were mainly detected at E7.5 and E8.5. Firstly, the cell annotation information and gene expression matrix were exported from the Seurat object with suggested scripts of the CellPhoneDB protocol. Then, the cell annotation information and count expression matrix were used as the input for the CellPhoneDB statistical analysis with default settings, and this step together with the following plotting step was executed in the Linux system as the protocol suggested. The database of receptor-ligand interactions was generated for human proteins, and the genes of the mouse have been transferred to human genes. Finally, we showed some significant interactions between TSCs and ExE cells and the remaining cell types at the mouse maternal-fetal interface with the CellPhoneDB dot plot function.

### Immunohistochemistry

Whole uteri (E7.5–E14.5) and dissected placentae (E12.5–E14.5) were fixed in a 4% paraformaldehyde (P6148, Sigma-Aldrich) solution and embedded in paraffin. Sections of 5 μm thickness were cut on a Leica paraffin microtome. After deparaffinization and rehydration, antigen retrieval was performed by boiling the sections in 10 mM sodium citrate buffer (pH 6.0), followed by blocking in 3% bovine serum albumin (A4503, Sigma-Aldrich). For immunohistochemistry, the primary antibody was used against cytokeratin (Z0622, Dako). Other procedures followed the user manual of the Two-Step IHC kit (ZSGB-Bio, SP9001). Nuclear counterstaining was performed with hematoxylin. Images were obtained under a Leica Aperio VESA8 microscope and processed with Aperio ImageScope software. For immunofluorescence analysis, the primary antibody against *Prl3d1* (SC-34713, Santa Cruz) was used at a 1:200 dilution, and incubated overnight. The secondary antibody used was donkey anti-goat Alexa Fluor 488 (A11055, Invitrogen). Nuclear counterstaining was performed with 4′,6-diamidino-2-phenylindole (DAPI) (10236276001, Millipore Sigma). Images were observed under a Zeiss LSM 780 confocal microscope and processed with ZEN software.

### RNAscope in situ hybridization

Formalin-fixed paraffin-embedded mouse uterine tissues (with the fetus and extraembryonic tissues) and placentae were subjected to in situ hybridization following user instructions of RNAscope Multiplex Fluorescent Reagent Kit V2 Assay and RNA Protein Co Detection Assay (Advanced Cell Diagnostics Bio). Hybridization was performed with RNAscope probes Mm-Cdx2 (438921), Mm-Hand1-C2 (429651-C2), Mm-Ovol2 (558501), Mm-Msx2 (421851-C3), Mm-Bhlhe40 (467651), Mm-Cdx1 (583561), Mm-Bhlhe41 (467431), and Mm-Foxo4 (503791); RNAscope probe 2-plex positive control probe-Mouse (320761); and RNAscope probe 2-plex negative control probe (320751). RNA and protein co-detection assay was performed with antibodies against Cytokeratin (Z0622, Dako, 1:100), CTSQ (ab171840, Abcam, 1:100), and MCT4 (AB3314P, Millipore Sigma, 1:100). Nuclei were stained with 4′,6-diamidino-2-phenylindole (DAPI) (10236276001, Millipore Sigma). Images were obtained under a Zeiss LSM 780 confocal microscope and processed with ZEN software.

### Cell culture

The mTSC line isolated from CD1 mouse blastocysts (a kind gift from the Zhou lab, Beijing, China) was grown as previously described (15). mTSC culture conditions: 20% fetal bovine serum (FBS) (Gibco, 10091148), 1 mM sodium pyruvate (Gibco, 11360070), 50 mg/mL penicillin-streptomycin solution (Gibco, 15070063), 0.1 mM 2-mercaptoethanol (Gibco, 31350), 25 ng/mL recombinant human FGF4 (Peprotech, 100-31), and 1 μg/mL heparin (Sigma, H3149) in RPMI 1640 with L-glutamine (Gibco, 11875119), with 70% of the medium pre-conditioned on mouse embryonic fibroblasts (CM). The medium was changed every 2 days, and the cells were passaged until they reached confluency. Trypsinization (0.25% trypsin/EDTA) was carried out at 37 °C for approximately 5 min. The differentiation medium consisted of unconditioned TSC medium without recombinant human FGF4 and heparin.

### Gene-KD using siRNA

For gene-KD, 3 × 10^5^ cells were transfected with 50 nM siRNA with jetPRIME transfection reagent (Polyplus, 101000027) as recommended by the manufacturer. The applied siRNA sequences are provided in Supplementary Table S5.

### RT-qPCR

Total RNA was extracted using TRIzol reagent (Gibco, 15596018), and cDNA synthesis was performed with HiScript II reverse transcriptase (Vazyme, R201). Quantitative (q) PCR was performed using TB Green Fast qPCR Mix (Takara, RR430A) and primer pairs (Supplementary Table S5) on a Roche LightCycler 480 System. Normalized expression levels are displayed as the mean relative to the negative control sample; error bars indicate standard errors of the means (SEM) of at least three replicates. Where appropriate, Student’s *t* tests were performed to calculate statistical significance of expression differences (*P* < 0.05) using GraphPad Prism 9.

## Supplementary information

Supplementary Figures

Table S1

Table S2

Table S3

Table S4

Table S5

## Data Availability

The data reported in this paper are available at the NCBI Gene Expression Omnibus (GEO) under the accession number GSE156125. The main codes employed for the analysis are available at GitHub (https://github.com/jxxlab/mouse_placenta). All other relevant data and materials in this study are available from the corresponding authors upon reasonable request.
