# Supplementary material for: A differentiation roadmap of murine placentation at single-cell resolution
Source: Cell Discov. 2023 Mar 17;9:30. doi: 10.1038/s41421-022-00513-z (PMC10020559; doi:10.1038/s41421-022-00513-z)
Supplement: Supplementary file 1 — Supplementary Figures [file 41421_2022_513_MOESM1_ESM.pdf]

Supplementary Figure S1

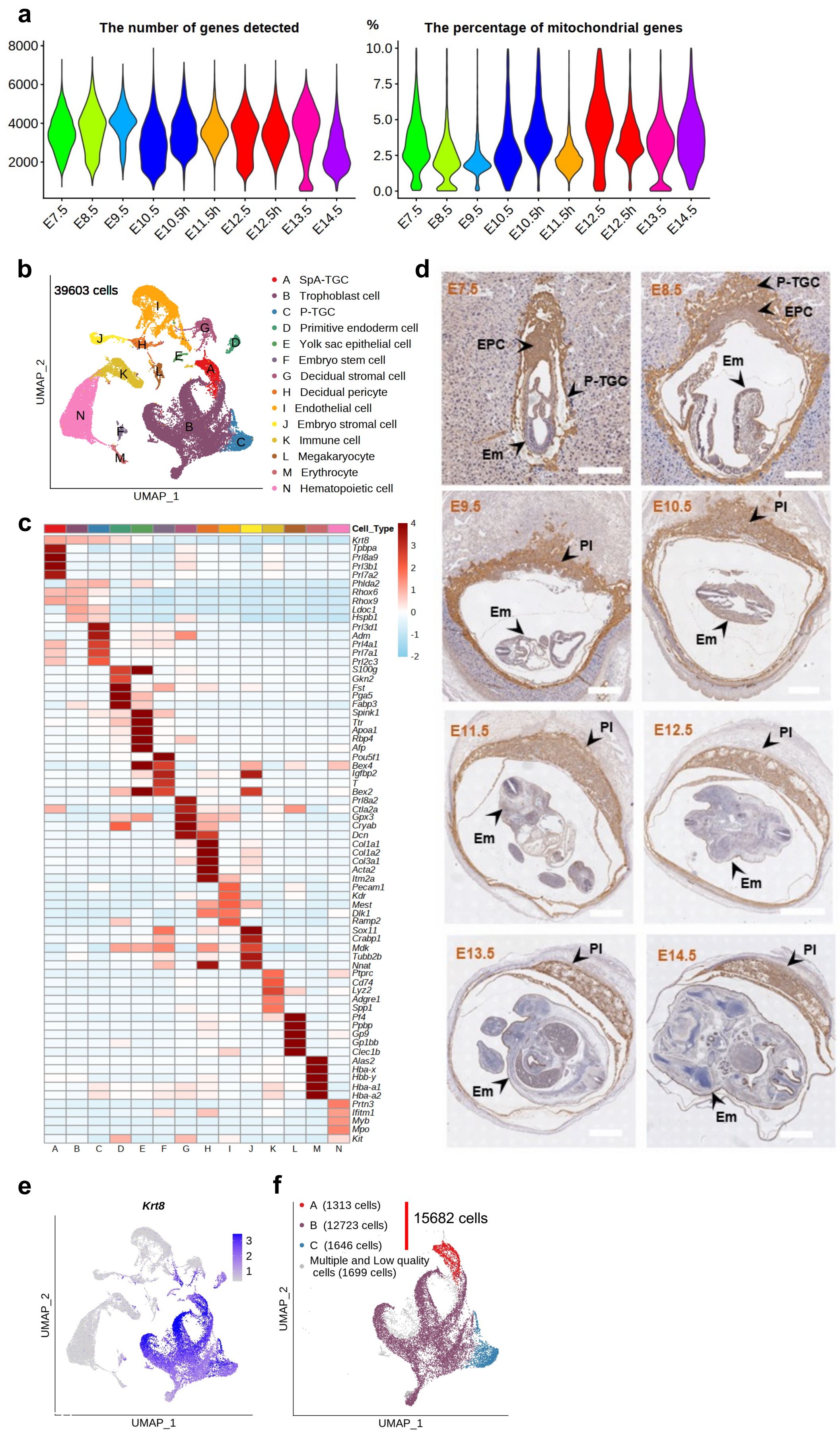

Supplementary Fig. S1. The filtering of mouse trophoblast cells.

(**a**), Violin plots showing the number of genes detected (left) and the percentage of mitochondria genes (right) per cell per sample. Letter h represents the data from another study (GSE152903) of us associated with placental hematopoiesis.

(**b**), Expression matrix-based UMAP plot showing all of the captured cells isolated from mouse placentae (39603 cells) after the unsupervised clustering step. Cells are colored by cell clusters.

(**c**), Heat map depicting the representative differentially expressed genes for the main cell types. The color key from light blue to red indicates low to high gene expression level.

(**d**), Representative stains for the Keratin (the marker of epithelial cells). Scale bars: 300 μm (E7.5, E8.5); 500 μm (E9.5); 700 μm (E10.5); 1mm (E11.5); 2 mm (E12.5, E13.5, E14.5). EPC, ectoplacental cone; P-TGC, parietal trophoblast giant cell; Pl, placenta; Em, embryo.

(**e**), UMAP plot as shown in (**b**) showing the expression of *Krt8*.

(**f**), UMAP plot separated from (**b**), with cells colored by cell clusters and other features after further annotating for multiple cells and low quality cells.

Supplementary Figure S2

a

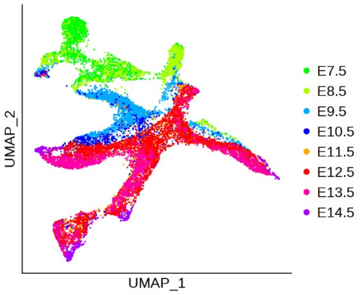

b

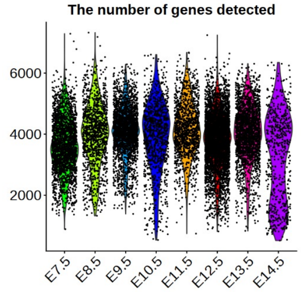

c

Embryonic day	7.5	8.5	9.5	10.5	10.5h	11.5h	12.5	12.5h	13.5	14.5	Total
Extraembryonic tissues/ Placentae	6+7	7	8	8	8	8	6	7	7	7	79
Cell viabilities	93%	92%	90%	88%	92%	90%	91%	86%	82%	76%	NA
Pregnancies	2	1	1	1	1	1	1	1	1	1	11
10x libraries	1	1	1	1	1	1	1	1	1	1	10
No. of batch	1	1	2	3	5	6	3	6	2	4	6
Cells (before filtering)	3259	1880	2808	2950	2885	8245	5842	3371	3403	4960	39603
Trophoblast Cells (after filtering)	2311	1218	2468	286	714	1301	2573	2081	2130	600	15682

d

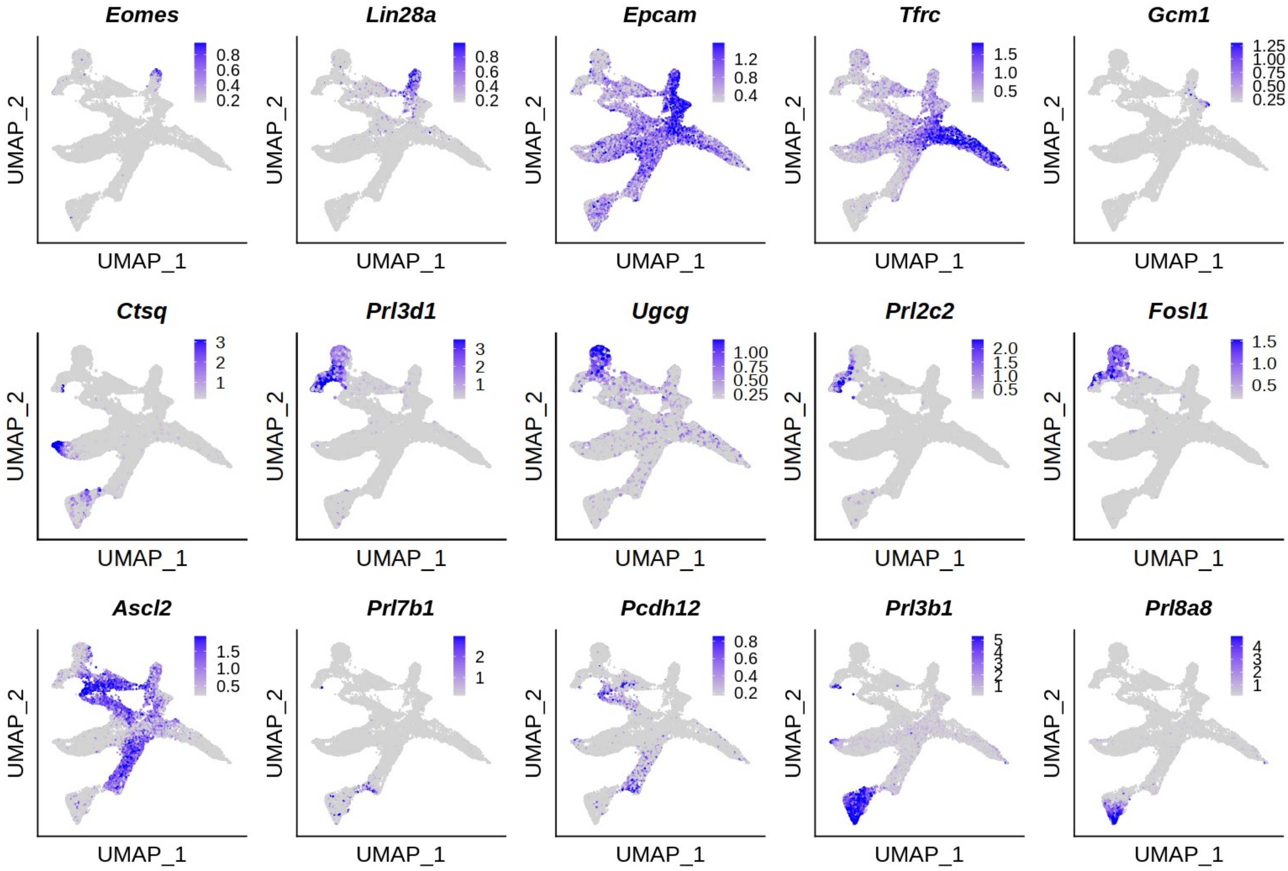

Supplementary Fig. S2. The annotation of mouse trophoblast cells.

(**a**), UMAP plot as shown in **Fig. 1b**, with cells colored by time points of sample collection.

(**b**), Violin plot showing the detected gene numbers of the filtered 15682 mouse trophoblast cells per cell at indicated sampling time point.

(**c**), Table showing the detailed information about sample collection and sequencing library construction. Letter h represents the data from another study (GSE152903) of us associated with placental hematopoiesis.

(**d**), UMAP plots as shown in **Fig. 1b**, showing the expression of specific marker genes for main cell clusters.

Supplementary Figure S3

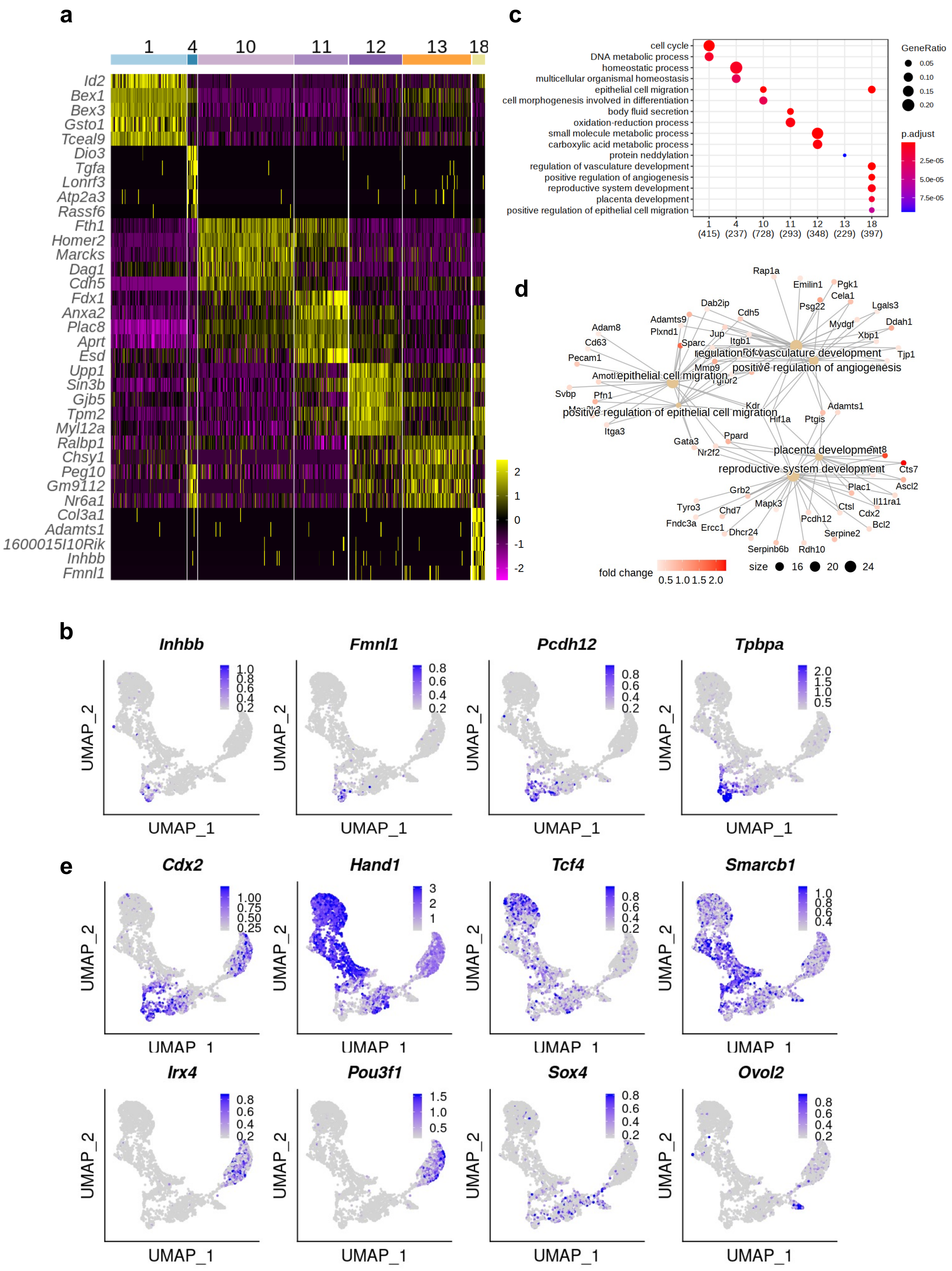

Supplementary Fig. S3. Analyses of mouse trophoblast cells before chorioallantoic fusion.

(**a**), Heat map showing top 5 highly expressed genes for each cell cluster shown in **Fig. 2a.** Yellow to purple corresponds to high to low expression levels.

(**b**), UMAP plots as shown in **Fig. 2a** (middle) showing the highly expressed genes in cluster 18.

(**c**), The Gene Ontology (GO, Biological Process) results enriched with highly expressed genes of cell clusters at E7.5-E8.5. The scaled significance (adjusted *P* value) from low to high is presented as color scale from blue to red. The size of the dots represents the percentage of genes for each GO category.

(**d**), Network diagram showing 6 representative GO categories that enriched with highly expressed genes of EPC migratory cells. The node size indicates the enriched counts of genes for the gene ontology categories, and the node color of genes indicates the fold change of gene expression level in EPC migratory cells compared with remaining trophoblast cells.

(**e**), UMAP plot as shown in **Fig. 2a** (middle) showing the expression of TFs indicated.

Supplementary Figure S4

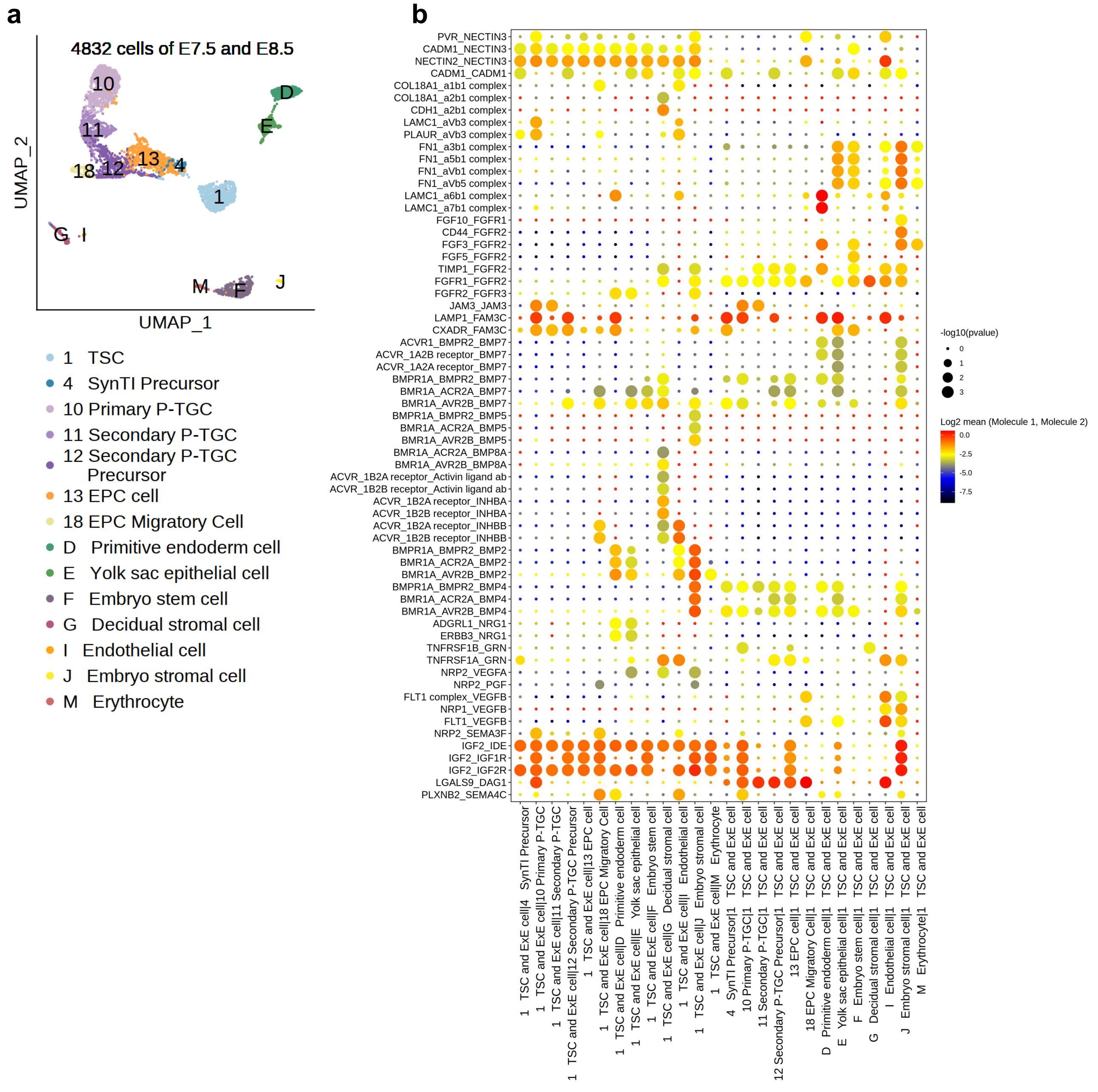

Supplementary Fig. S4. The cell-cell communication analysis for TSC and ExE cells.

(**a**), UMAP plot produced with all the captured cells at E7.5 and E8.5 by the Seurat flow. Cells are colored by cell clusters as indicated.

(**b**), Dot plot showing predicted signaling interactions between cluster 1 (TSC and ExE cell) and other cell types. *p*-values are indicated by circle size, scale on right. The means of the average expression level of interacting molecule 1 in cluster 1 and interacting molecule 2 in cluster 2 are indicated by color.

Supplementary Figure S5

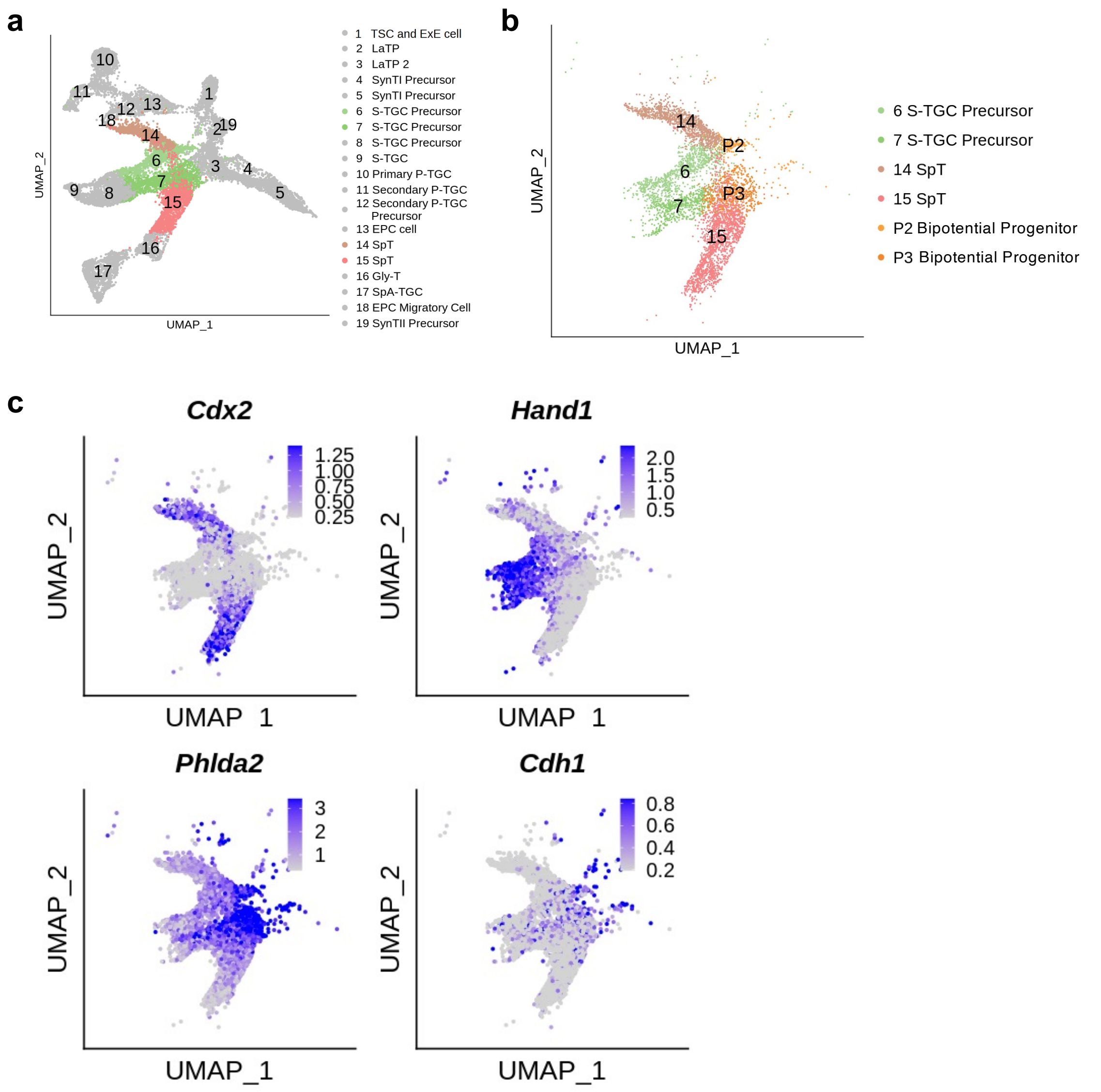

Supplementary Fig. S5. The analysis of bipotential progenitor cells.

(**a**), UMAP plot as shown in **Fig. 1b**, with cells colored by cell clusters.

(**b**), UMAP plot separated from (**a**), with cells colored by cell clusters shown as indicated.

(**c**), UMAP plot as shown in (**b**) showing the expression of highly expressed genes in cluster P1, E1, and E2.

Supplementary Figure S6

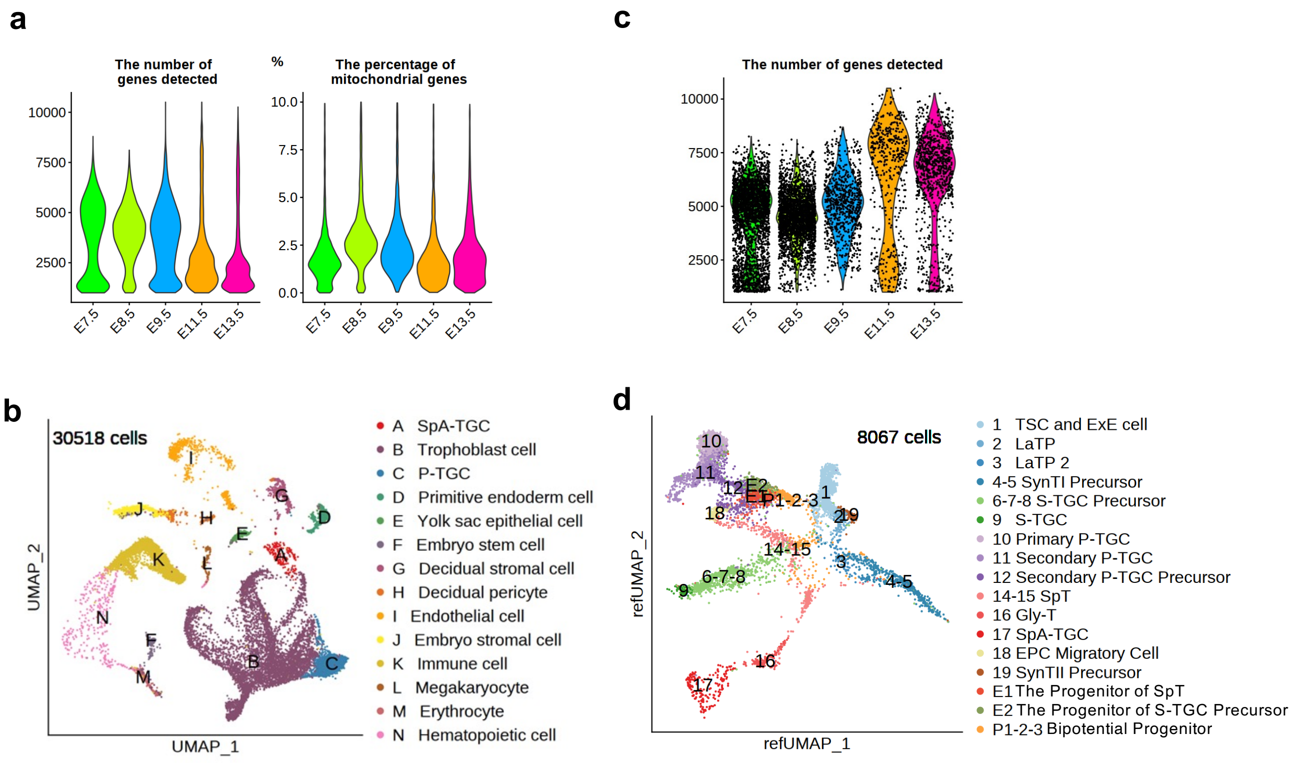

e

Embryonic day	7.5	8.5	9.5	11.5	13.5	Total
Extraembryonic tissues/ Placentae	9	9	10	8	8	44
Cell viabilities	96.2%	87.6%	81.1%	57.3%	76.8%	NA
Pregnancies	1	1	1	1	1	5
10x libraries	1	1	1	1	1	5
No. of batch	1	2	3	4	5	5
Cells (before filtering)	6060	5899	5619	6358	6582	30518
Trophoblast Cells (after filtering)	3056	2749	814	507	941	8067

Supplementary Fig. S6. The filtering, mapping, and annotating of 10× v3 single-cell data

(**a**), Violin plots showing the number of genes detected (left) and the percentage of mitochondria genes (right) per cell per sample sequenced with 10× v3 kit after excluding cells with less than 1000 detected genes and more than 10% mitochondrial genes.

(**b**), UMAP plot showing all the captured cell types (30518 cells) sequenced with 10× v3 kit after projecting the 10× v3 single-cell data onto the 10× v2 single-cell data based UMAP structure shown in **Supplementary Fig. S2b**. Cells are colored by cell clusters as indicated..

(**c**), Violin plot showing the detected gene numbers per cell per sample after retaining 8067 10× v3 trophoblast cells with high quality.

(**d**), UMAP plot showing 8067 mouse trophoblast cells after projecting the 10× v3 single-cell data of 8014 mouse trophoblast cells onto the 10× v2 single-cell data based UMAP structure shown in **Fig. 1b**. Cells are colored by cell clusters as indicated.

(**e**), Table showing the detailed information about sample collection and sequencing library construction with 10× v3 kit.

Supplementary Figure S7

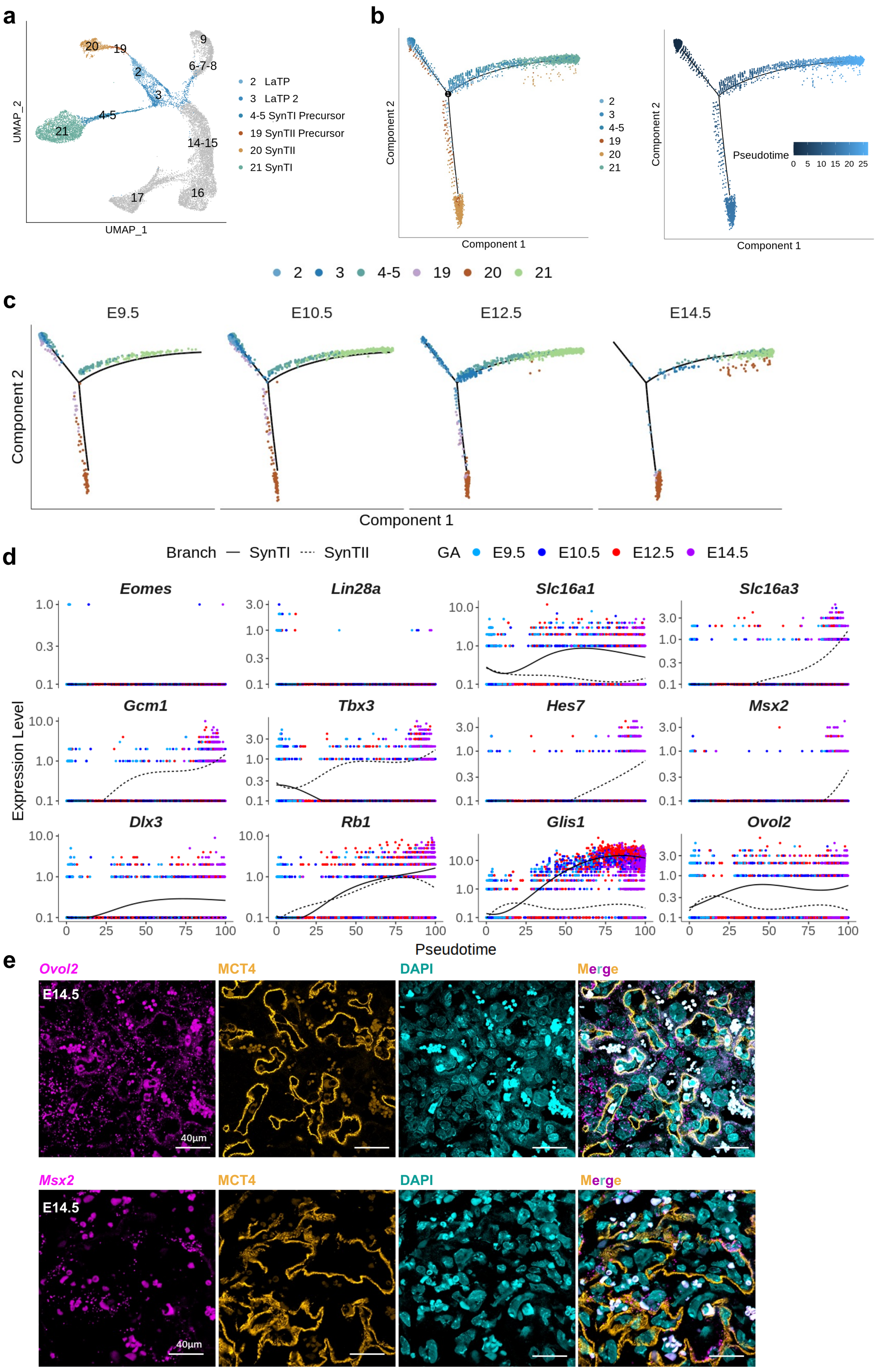

Supplementary Fig. S7. The analysis of chorion branch trophoblast cells using single nuclei RNA-seq data.

(**a**), UMAP plot showing chorion branch nuclei, with nuclei colored by clusters as indicated.

(**b**), Pseudotime ordering of nuclei annotated in (**a**), with nuclei colored by clusters (left) and pseudotimes (right, deep blue to light blue corresponds to developmental pseudotime from early to late) as indicated.

(**c**), Pseudotime trajectory plots splited by sampling time points from (**b**, left), with nuclei colored by clusters as indicated.

(**d**), Pseudotemporal kinetics plots showing the expression of representative TFs involved in chorion cell development. As shown on the top-left corner of the image, the full line indicates the dynamic expression of TFs in SynTI branch cells across developmental pseudotime; and the dashed line indicates the dynamic expression of TFs in SynTII branch cells across developmental pseudotime. Points are colored by the sampling time as indicated on the top-right corner of the image.

(**e**), Representative images of E14.5 mouse placenta sections probed for *Ovol2* (magenta) and *Msx2* (magenta) transcripts and co-stained for MCT4 protein (orange). DAPI, nucleus (cyan, here and after). Scale bars as indicated.

Supplementary Figure S8.

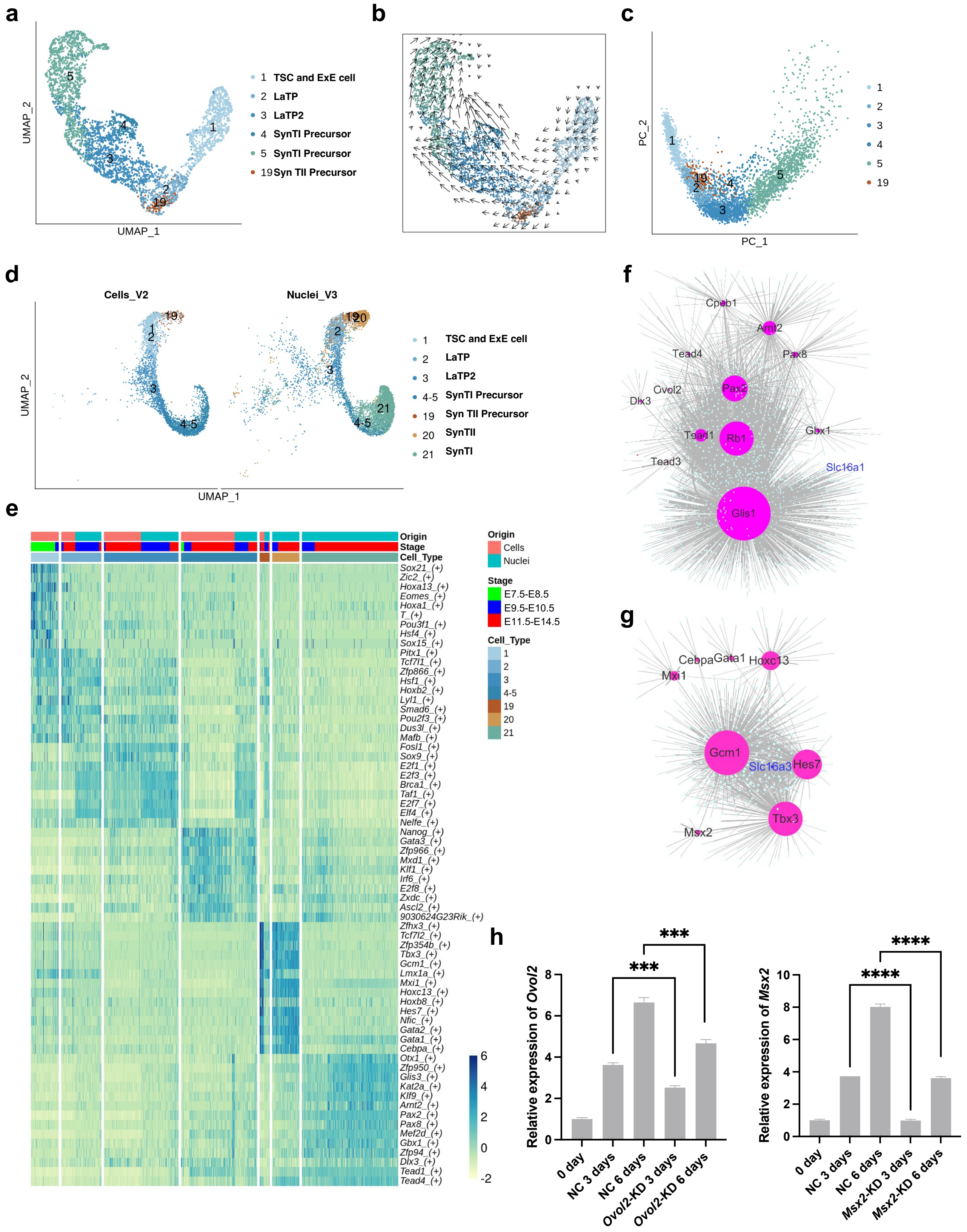

Supplementary Fig. S8. The differentiation of chorion branch trophoblast cells.

(**a**), UMAP plot reproduced with chorion branch cells by the Seurat flow. Cells are colored by cell clusters.

(**b**), RNA velocity field projected onto the UMAP plot shown in (**a**). Arrows show the local average velocity evaluated on a regular grid.

(**c**), PCA plot showing chorion branch trophoblast cells, with cells colored by cell clusters shown in (**a**) .

(**d**), UMAP plots showing the chorion branch cells (left) and nuclei (right).

(**e**), Heat map showing the activities of representative TFs with cells and nuclei shown in (**d**). The color key from yellow to blue indicates low to high TF activity. Cells and nuclei are colored by data origin, cell clusters, and sampling time.

(**f**), Regulatory network visualizing potential key TFs for the differentiation of SynTI cells. Nodes that are colored in magenta indicates TFs, and the downstream genes are nodes colored by light blue. The node size indicates the number of connections, and the line size indicates the weight of connection.

(**g**), Regulatory network visualizing potential key TFs for the differentiation of SynTII cells. Nodes that are colored in magenta indicates TFs, and the downstream genes are nodes colored by light blue. The node size indicates the number of connections, and the line size indicates the weight of connection.

(**h**), RT-qPCR analysis of the expression of *Ovol2* (left) and *Msx2* (right) in *Ovol2*-KD and *Msx2*-KD mTSCs compared to the negative control (NC, here and after). Data are expressed as the mean ± SEM of three independent replicates (*** $p < 0.001$, ****$p < 0.0001$, Student's *t*-test).

Supplementary Figure S9

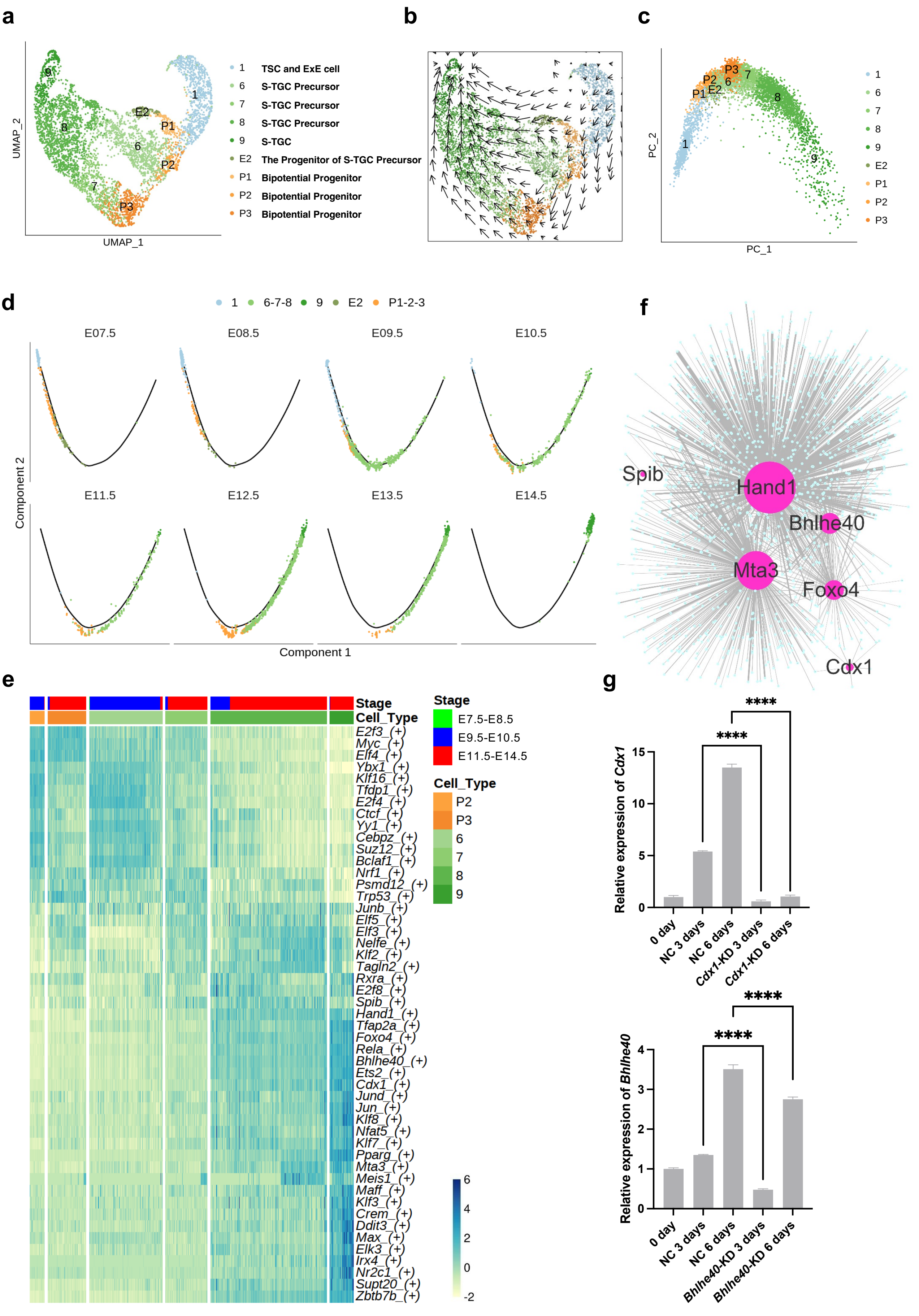

Supplementary Fig. S9. The differentiation of S-TGC branch trophoblast cells.

(**a**), UMAP plot reproduced with cells shown in **Fig. 6a** by the Seurat flow. Cells are colored by cell clusters.

(**b**), RNA velocity field projected onto the UMAP plot shown in (**a**). Arrows show the local average velocity evaluated on a regular grid.

(**c**), PCA plot showing S-TGC branch trophoblast cells, with cells colored by cell clusters shown in (**a**).

(**d**), Pseudotime trajectory plots split by sampling time points from **Fig. 6b**, with cells colored by clusters as indicated.

(**e**), Heat map showing the activities of representative TFs with cells belong to S-TGC branch. The color key from yellow to blue indicates low to high TF activity. Cells and nuclei are colored by cell clusters and sampling time.

(**f**), Regulatory network visualizing potential key TFs for the differentiation of S-TGCs. Nodes that are colored in magenta indicates TFs, and the downstream genes are nodes colored by light blue. The node size indicates the number of connections, and the line size indicates the weight of connection.

(**g**), RT-qPCR analysis of the expression of *Cdx1* (top) and *Bhlhe40* (bottom) in *Cdx1*-KD and *Bhlhe40*-KD mTSCs compared to the negative control. Data are expressed as the mean ± SEM of three independent replicates (****$p < 0.0001$, Student's *t*-test).

Supplementary Figure S10

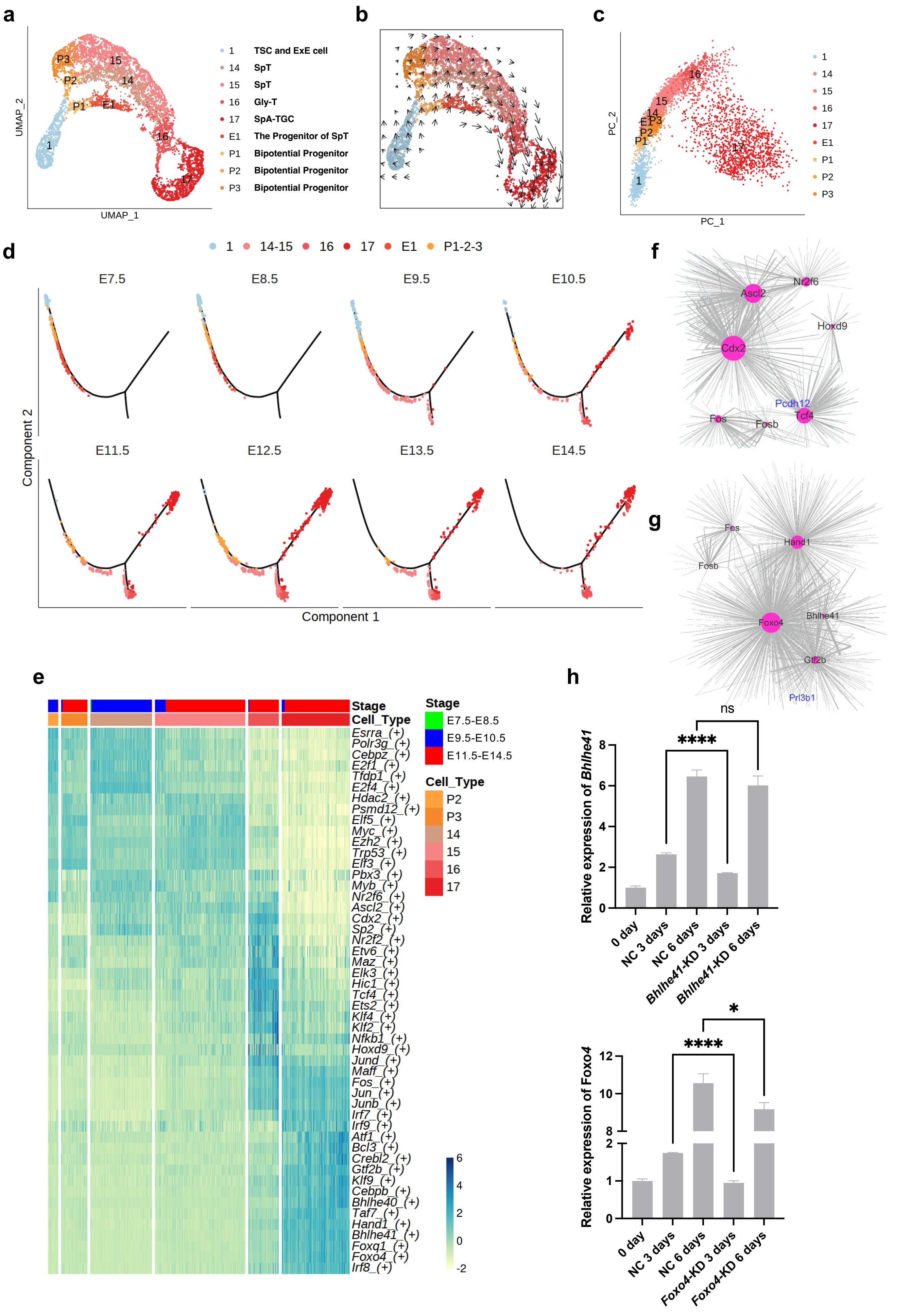

Supplementary Fig. S10. The differentiation of spongio-branch trophoblast cells.

(**a**), UMAP plot reproduced with cells shown in **Fig. 7a** by the Seurat flow. Cells are colored by cell clusters.

(**b**), RNA velocity field projected onto the UMAP plot shown in (**a**). Arrows show the local average velocity evaluated on a regular grid.

(**c**), PCA plot showing spongio-branch trophoblast cells, with cells colored by cell clusters shown in (**a**) .

(**d**), Pseudotime trajectory plots split by sampling time points from **Fig. 7b**, with cells colored by cell clusters as indicated.

(**e**), Heat map showing the activities of representative TFs with cells belong to spongio-branch. The color key from yellow to blue indicates low to high TF activity. Cells and nuclei are colored by cell clusters and sampling time.

(**f**), Regulatory network visualizing potential key TFs for the differentiation of Gly-T cells. Nodes that are colored in magenta indicates TFs, and the downstream genes are nodes colored by light blue. The node size indicates the number of connections, and the line size indicates the weight of connection.

(**g**), Regulatory network visualizing potential key TFs for the differentiation of SpA-TGCs. Nodes that are colored in magenta indicates TFs, and the downstream genes are nodes colored by light green. The node size indicates the number of connections, and the line size indicates the weight of connection.

(**h**), RT-qPCR analysis of the expression of *Bhlhe41* (top) and *Foxo4* (bottom) in *Bhlhe41*-KD and *Foxo4*-KD mTSCs compared to the negative control. Data are expressed as the mean ± SEM of three independent replicates (*$p < 0.05$, ****$p < 0.0001$, ns: not significant, Student's *t*-test).
